# Osteoblast-derived vesicles induce a switch from bone-formation to bone-resorption in vivo

**DOI:** 10.1038/s41467-022-28673-2

**Published:** 2022-02-24

**Authors:** Maki Uenaka, Erika Yamashita, Junichi Kikuta, Akito Morimoto, Tomoka Ao, Hiroki Mizuno, Masayuki Furuya, Tetsuo Hasegawa, Hiroyuki Tsukazaki, Takao Sudo, Keizo Nishikawa, Daisuke Okuzaki, Daisuke Motooka, Nobuyoshi Kosaka, Fuminori Sugihara, Thomas Boettger, Thomas Braun, Takahiro Ochiya, Masaru Ishii

**Affiliations:** 1 Department of Immunology and Cell Biology, Graduate School of Medicine and Frontier Biosciences, Osaka University, Suita, Osaka Japan; 2 WPI-Immunology Frontier Research Center, Osaka University, Suita, Osaka Japan; 3 Laboratory of Bioimaging and Drug Discovery, National Institutes of Biomedical Innovation, Health and Nutrition, Ibaraki, Osaka Japan; 4 Genome Information Research Center, Research Institute for Microbial Diseases, Osaka University, Suita, Osaka Japan; 5 Division of Molecular and Cellular Medicine, Tokyo Medical University, Shinjuku, Tokyo Japan; 6 Core Instrumentation Facility, Immunology Frontier Research Center and Research Institute for Microbial Diseases, Osaka University, Suita, Osaka Japan; 7 Max-Plank-Institute for Heart and Lung Research, Bad Nauheim, Germany

**Keywords:** Osteoclasts, Multiphoton microscopy, miRNAs, Bone, Osteoblasts

## Abstract

Bone metabolism is regulated by the cooperative activity between bone-forming osteoblasts and bone-resorbing osteoclasts. However, the mechanisms mediating the switch between the osteoblastic and osteoclastic phases have not been fully elucidated. Here, we identify a specific subset of mature osteoblast-derived extracellular vesicles that inhibit bone formation and enhance osteoclastogenesis. Intravital imaging reveals that mature osteoblasts secrete and capture extracellular vesicles, referred to as small osteoblast vesicles (SOVs). Co-culture experiments demonstrate that SOVs suppress osteoblast differentiation and enhance the expression of receptor activator of NF-κB ligand, thereby inducing osteoclast differentiation. We also elucidate that the SOV-enriched microRNA miR-143 inhibits Runt-related transcription factor 2, a master regulator of osteoblastogenesis, by targeting the mRNA expression of its dimerization partner, core-binding factor β. In summary, we identify SOVs as a mode of cell-to-cell communication, controlling the dynamic transition from bone-forming to bone-resorbing phases in vivo.

## Abstract (web summary)

Bone remodeling involves a switch between bone formation and resorption, but the mechanisms is unclear. Here, the authors show that intercellular communication via extracellular vesicles secreted by mature osteoblasts is a key factor for the switching, via a microRNA-mediated mechanism.

## Introduction

Bone remodeling occurs in different parts of the body throughout life to maintain bone architecture balance and systemic mineral homeostasis. During this “remodeling” process, osteoclasts remove mineralized bones, whereas osteoblasts form new bones^1–3^. These resorption and formation phases are linked and balanced with intermittent coupling phases. Functional coupling between these two cell types is critical for the maintenance of proper bone metabolism, and the mechanisms controlling the transition from bone-resorbing to bone-forming phases (i.e., reversal phase) have been investigated^4–6^. These mechanisms are regulated by insulin-like growth factor I and transforming growth factor-β released from the bone matrix during bone resorption^5^, sphingosine-1-phosphate^7^, collagen triple helix repeat containing 1^8^, Semaphorin 4d^9^, ephrin B2^10^, and receptor activator of NF-κB ligand (RANKL)/RANK reverse signaling^11^. Notably, all of these factors contribute to the transition from bone-resorbing to bone-forming phases. Nevertheless, the molecular and cellular mechanisms terminating osteoblastic bone formation and promoting osteoclastic bone resorption (i.e., “reciprocal” reversal phase) remain elusive.

Intravital optical imaging using multiphoton microscopy can help dissect in vivo cellular dynamics in various intact tissues and organs^12–14^. To understand the spatiotemporal dynamics of bone remodeling in vivo, we established an intravital imaging technique to visualize the intact bone tissues of living mice^15–18^. Using this method, we explored the interplay between bone-destroying osteoclasts and bone-forming osteoblasts and found that direct cell-to-cell contact led to the inhibition of osteoclastic bone resorption^19^. However, the spatial resolution of intravital bone imaging was insufficient to visualize structures smaller than cells.

In this study, we established an advanced high-resolution microscopy system to visualize extracellular vesicles secreted and captured by mature osteoblasts (mOBs) in vivo. We identified a subset of mOB-derived vesicles limiting bone formation and stimulating osteoclastogenesis, thus regulating the “reciprocal reversal phase,” through a microRNA (miRNA)-mediated mechanism.

## Results

### Visualization of small osteoblast-derived vesicles

**Fig. 1:**
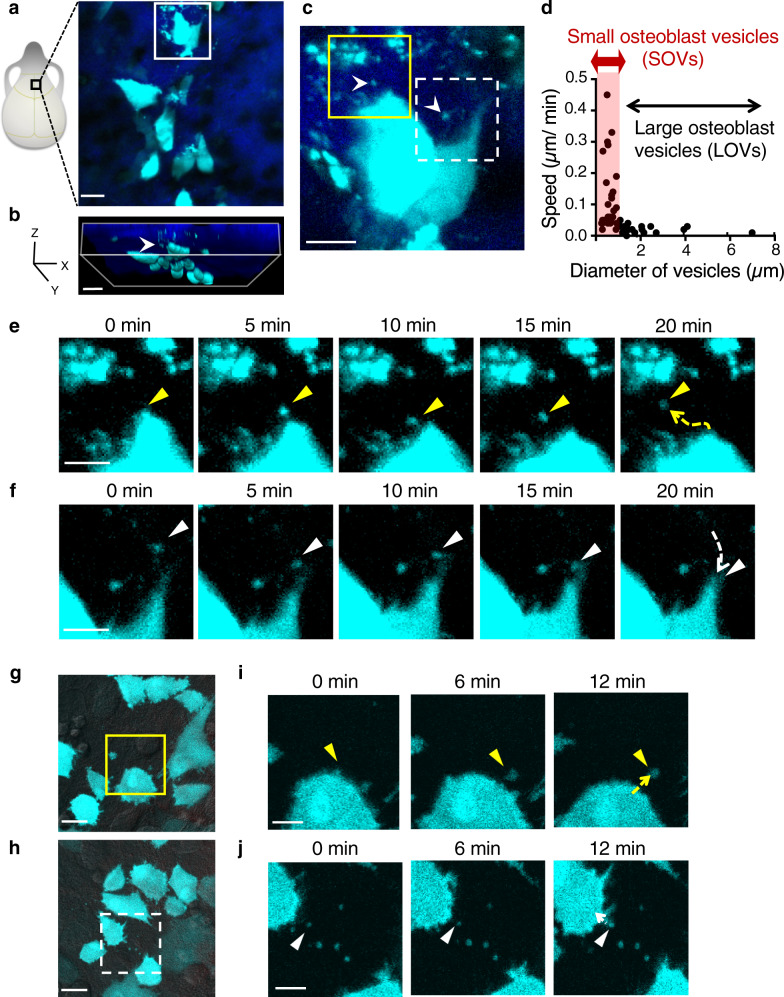
Communication among osteoblasts via small osteoblast vesicles in vivo and in vitro. **a** Schematic representation of mouse skull bone (left) and an intravital multiphoton, maximum-intensity projection image of skull bone tissues from Col2.3-ECFP mice (a representative image, at least three independent experiments) (right). Cyan: mature osteoblasts (mOBs) and extracellular vesicles (EVs) from mOBs; blue: bone surface, second harmonic generation. Scale bar: 20 μm. **b** Three-dimensional image of visual field shown in (**a**). Arrowhead indicates an EV. Scale bar: 20 μm. **c** Magnified view of region outlined in (**a**). Arrowheads indicate EVs. Scale bar: 10 μm. **d** Speed of mOB-derived EVs by tracking analysis of EVs shown in (**c**) (*n* = 51). SOVs: small osteoblast vesicles with a diameter of ≤1 µm; LOVs: large osteoblast vesicles with a diameter of >1 µm. **e** Consecutive time-lapse images of SOVs released by mOBs indicated by yellow lines in (**c**). Arrowheads and dotted lines indicate SOVs and their tracks, respectively. Scale bar: 5 µm. **f** Consecutive time-lapse images of SOVs taken up by mOBs indicated by dotted lines in (**c**). Arrowheads and dotted lines indicate SOVs and their tracks, respectively. Scale bar: 5 µm. **g**, **h** Representative images of primary osteoblasts of Col2.3-ECFP mice of at least three independent experiments. Cyan: mOBs and mOB-derived EVs. Scale bar: 20 µm. **i** Consecutive time-lapse images of released SOVs indicated by yellow lines in (**g**). Arrowheads and dotted lines indicate SOVs and their tracks, respectively. Scale bar: 10 µm. **j** Consecutive time-lapse images of internalized SOVs indicated by dotted lines in (**h**). Arrowheads and dotted lines indicate SOVs and their tracks, respectively. Scale bar: 10 µm.

We have previously generated reporter mice expressing enhanced cyan fluorescent protein (ECFP) in mOBs driven by a 2.3 kb fragment of rat type I collagen α promoter (Col1a1*2.3) (Col2.3-ECFP mice). In this reporter line, ECFP is expressed in bone-forming mOBs but not in immature osteoblasts^19^. We performed intravital multiphoton imaging using high spatial resolution to visualize bone tissues in Col2.3-ECFP mice, and observed many ECFP-positive vesicles in the vicinity of the cell bodies of mOBs (Fig. 1a–c). Tracking analyses showed that the small osteoblast vesicles (SOVs) had a diameter of ≤1 µm and had a higher speed than large osteoblast vesicles (LOVs), which were >1 µm in diameter (Fig. 1d). Moreover, time-lapse imaging revealed that SOVs were released to the extracellular space and were taken up by mOBs in vivo within ~20 min (Fig. 1e, f; Supplementary Video 1). SOV release and uptake were also observed in vitro in primary osteoblasts from Col2.3-ECFP mice (Fig. 1g–j; Supplementary Videos 2, 3). The uptake of SOVs by mOBs was confirmed by co-culture of mOBs and membrane-labeled SOVs (Supplementary Fig. S1a–c; Supplementary Videos 4, 5). We also found that SOVs expressed CD63, a marker of extracellular vesicles^20^ (Supplementary Fig. S1d, e). These results suggest that mOBs actively secrete and take up SOVs both in vivo and in vitro, communicating via extracellular SOVs in a paracrine or autocrine manner.

### SOVs of approximately 400 nm in diameter suppress osteoblast differentiation

**Fig. 2:**
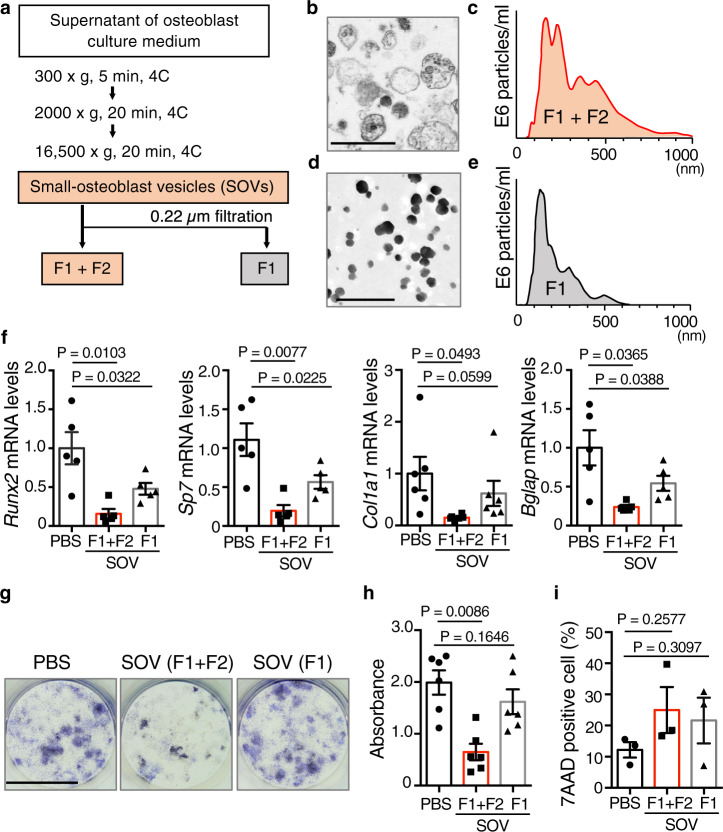
SOV-F2 inhibits osteoblast differentiation. **a** Schematic representation of SOV isolation via differential centrifugation. F1: fraction 1; F2: fraction 2. **b** Transmission electron micrograph of total SOVs (at least three independent fields from 1 mouse). Scale bar: 1 µm. **c** Nanoparticle tracking analysis of total SOVs (at least three independent experiments). **d** Transmission electron micrograph of SOV-F1 after 0.22 µm filtration (at least three independent fields from 1 mouse). Scale bar: 500 nm. **e** Nanoparticle tracking analysis of SOV-F1 after 0.22 µm filtration (at least three independent experiments). **f** *Runx2*, *Sp7*, *Col1a1*, and *Bglap* mRNA levels in osteoblasts treated with PBS, total SOVs (F1 + F2), or SOV-F1 (*Runx2*, *Sp7* and *Bglap*: *n* = 5; *Col1a1*: *n* = 6 biological replicates per group). **g**, **h** ALP activity in osteoblasts treated with PBS, total SOVs (F1 + F2), or SOV-F1 (*n* = 6 biological replicates per group). Representative images (**g**) and quantitative analysis (**h**) Scale bar: 10 mm. **i** Percentages of 7-AAD-positive apoptotic cells among CD45-negative primary osteoblasts at 6 days after treatment with PBS, total SOVs (F1 + F2), or SOV-F1 (*n* = 3 biological replicates per group). Data are means ± SEMs. Statistical significance was determined by one-way ANOVA with Dunnett’s multiple comparison post hoc test (**f**, **h**, **i**).

To characterize SOVs, we performed electron microscopy and nanoparticle tracking analyses to evaluate the morphology and size distribution of isolated SOVs from primary osteoblasts (Fig. 2a–e). Electron microscopy showed that the diameter of SOVs ranged from 100 nm to 1 µm, and that the structures and electron densities of SOVs varied (Fig. 2b). Nanoparticle tracking analyses revealed two peaks associated with different SOV sizes: one around 200 nm in diameter (hereafter called SOV-F1) and one around 400 nm in diameter (hereafter called SOV-F2) (Fig. 2c). SOV-F1 were isolated by 0.22-µm filtration (Fig. 2a); the collected SOV-F1 had similar sizes and electron densities (Fig. 2d, e). As the SOVs observed by intravital imaging ranged in size from 0.26 to 1 µm, they were likely SOV-F2.

Extracellular vesicles are composed of a lipid bilayer containing transmembrane proteins, as well as enclosed cytosolic proteins and RNAs (especially mRNAs and miRNAs). Extracellular vesicles are thought to serve as a mode of communication among neighboring or distant cells^21,22^. Here, we investigated the role of SOVs in the communication of neighboring osteoblasts. The relative expression levels of genes related to osteoblast differentiation in primary osteoblasts were analyzed 2 days after treatment with phosphate-buffered saline (PBS), total SOVs (F1 + F2), or SOV-F1 (Fig. 2f). The expression levels of *runt-related transcription factor 2* (*Runx2)* and *Osterix* (also known as *Sp7*), master transcription factors involved in osteoblast differentiation^23–25^, as well as those of the osteoblastic matrix proteins type 1 collagen (*Col1a1*) and osteocalcin (also known as *Bglap*), were significantly lower in the SOV-treated group than in the PBS control group (Fig. 2f). Total SOVs (F1 + F2) were more potent than SOV-F1 in suppressing the expression of osteoblast-related genes, suggesting that large SOVs mediate this transcriptional regulation. Consistently, in the osteoblastic cell line MC3T3-E1, the relative mRNA levels of *Runx2* and *Sp7* were significantly lower in the total SOV-treated group than in the PBS control group in a dose-dependent manner (Supplementary Fig. S2a). SOV-F1 treatment moderately affected the expression of *Runx2* but not the expression of *Sp7* (Supplementary Fig. S2b). To exclude the effects of proteins and RNAs outside the extracellular vesicles, such as membrane fragments and anchor proteins on the SOV membrane, we examined whether proteinase K and/or RNase treatment modulated the effects of SOVs on *Runx2* and *Sp7* expression. Inhibition by SOVs was unaffected by RNase and proteinase K (Supplementary Fig. S2c)^26^, indicating that components in SOVs, but not protein and RNA contaminants outside SOVs, were responsible for the inhibition of *Runx2* and *Sp7* expression.

We performed alkaline phosphatase (ALP; *Alpl*) staining to assess bone-forming activity in SOV-treated cells. ALP activity was significantly suppressed in the total SOV-treated group but not in the SOV-F1-treated group (Fig. 2g, h). We also assessed collagen production and mineralization by SOV-treated osteoblasts by Fast Green/Sirius Red staining and Alizarin Red S staining, respectively. Collagen production and mineralization by total SOV-treated osteoblasts were inhibited, compared with PBS control and SOV-F1-treated osteoblasts (Supplementary Fig. S2d, e). Next, we analyzed the effect of SOVs on cell death using 7-amino actinomycin D (7-AAD) staining; however, we found no significant differences among the groups (Fig. 2i). These results suggest that total SOVs (F1 + F2), but not SOV-F1, suppress osteoblast differentiation without affecting cell viability.

### SOV-treated osteoblasts promote osteoclastogenesis

**Fig. 3:**
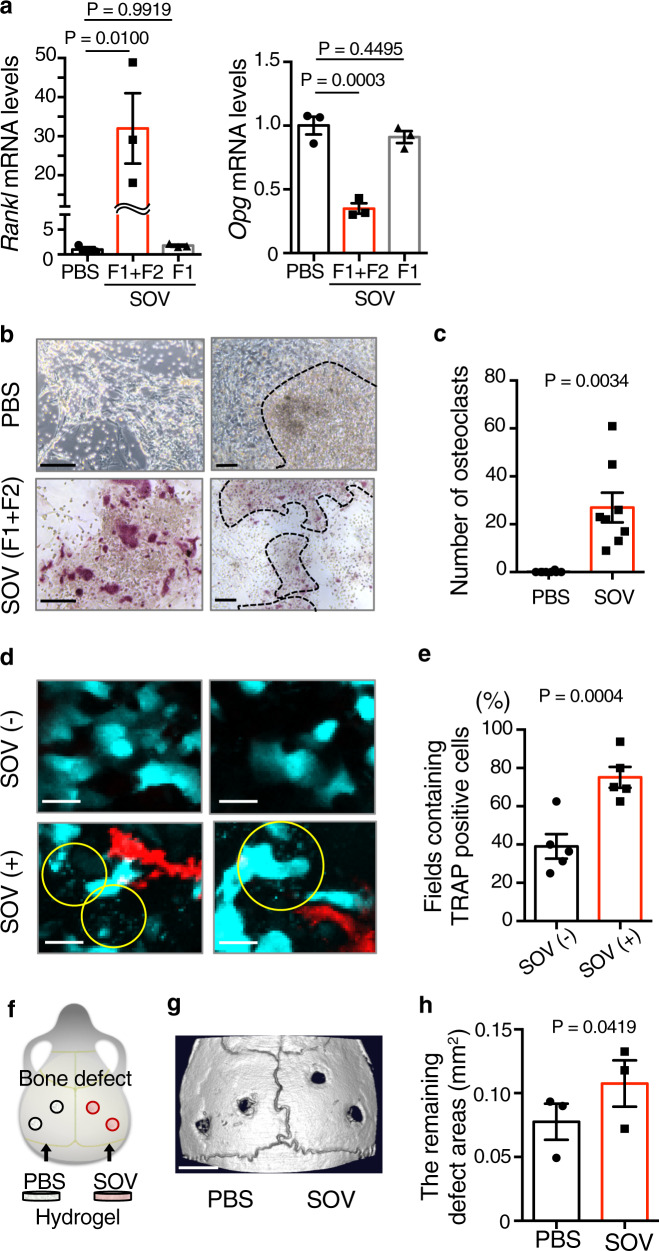
SOV-F2-treated osteoblasts induce osteoclastogenesis. **a** *Rankl* and *Opg* mRNA levels in primary osteoblasts after treatment with PBS, total SOVs (F1 + F2), or SOV-F1 (*n* = 3 biological replicates per group). **b** Representative image of TRAP-stained osteoclasts among BMMs co-cultured with PBS-treated osteoblasts (upper) or total SOV-treated osteoblasts (lower) (*n* = 8 independent experiments per group). Dotted lines at right represent nodules created by osteoblasts. Scale bar: 100 µm. **c** Number of osteoclasts among BMMs co-cultured with PBS-treated osteoblasts or total SOV-treated osteoblasts per well (*n* = 8 independent experiments per group). TRAP-positive multinucleated cells were counted as osteoclasts. **d** Representative in vivo images of calvaria from Col2.3-ECFP/TRAP-tdTomato mice of at least three independent experiments. Cyan: mature osteoblasts (mOBs) or SOVs; Red: mature osteoclasts (mOCs). Yellow circles show SOVs. mOCs were in the vicinity of mOBs secreting SOVs. Scale bar: 20 µm. **e** Percentages of areas where TRAP-positive cells overlapped with mOBs (*n* = 5 biological replicates per group). SOV(−): fields without SOVs; SOV(+): fields with SOVs. **f** Transplantation model of calvarial defects. Artificial bone defects on both sides of mouse calvaria were treated with hydrogels impregnated with PBS or total SOVs. **g** Representative micro-CT image of calvarial defects at 8 weeks after treatment with PBS or total SOVs. Scale bar: 1000 µm (*n* = 3 biological replicates per group). **h** Remaining calvaria defect areas at 8 weeks after treatment with PBS or total SOVs. Each point is the average area of two defects in each group (*n* = 3 biological replicates per group). Data are means ± SEMs. Statistical significance was determined by one-way ANOVA with Dunnett’s multiple comparison *post hoc* test in (**a**), two-tailed Welch’s *t*-test in (**c**), and two-tailed paired *t*-test in (**e**, **h**).

Osteoblast-lineage cells produce RANKL, encoded by the *Tnfsf11* (also known as *Rankl*) gene, an essential factor for osteoclastogenesis^27,28^, and osteoprotegerin (*Opg*), a decoy receptor for RANKL^28,29^, to regulate osteoclast differentiation and function. Therefore, we investigated the roles of SOVs in the expression of *Rankl* and *Opg*. The expression level of *Rankl* was significantly higher in the total SOV-treated group than in the PBS control group. In addition, the expression level of *Opg* was significantly lower in the total SOV-treated group than in the PBS control group. However, SOV-F1 did not affect *Rankl* or *Opg* expression (Fig. 3a). These results suggested an increased osteoclastogenic potential in total SOV-treated mOBs. Therefore, we assessed osteoclast differentiation using bone marrow macrophages (BMMs) co-cultured with either total SOV-treated osteoblasts or PBS-treated osteoblasts. When starting the co-culture, we washed out SOVs with culture medium to prevent SOVs from directly affecting BMMs. Tartrate-resistant acid phosphatase (TRAP)-positive mature osteoclasts (mOCs) were generated within 3 days of co-culture of BMMs with total SOV-treated osteoblasts without additional supplementation of recombinant RANKL. In contrast, no significant mOC generation was observed when BMMs were co-cultured with PBS-treated osteoblasts (Fig. 3b, c). To confirm the promotion of osteoclastogenesis by SOV-treated osteoblasts, we evaluated the expression levels of mOC marker genes: *Ctsk*, *Acp5*, and *Atp6v0d2*. The expression levels of all mOC marker genes were significantly higher in BMMs co-cultured with total SOV-treated osteoblasts than in BMMs co-cultured with PBS-treated osteoblasts (Supplementary Fig. S3a). Moreover, mOCs were exclusively induced on the nodules created by total SOV-treated osteoblasts in this co-culture system (Fig. 3b), indicating that SOVs serve as a local regulator of osteoclastogenesis by regulating osteoblasts. To exclude the direct effect of SOVs on BMMs, we cultured BMMs with SOVs in the presence or absence of macrophage colony-stimulating factor (M-CSF) without osteoblasts. At 3 and 7 days after SOV treatment, we evaluated osteoclast differentiation by TRAP staining. Both PBS-treated and SOV-treated BMMs did not differentiate into mOCs within 3 or 7 days, irrespective of the presence of M-CSF (Supplementary Fig. S3b). Therefore, we concluded that SOVs do not independently affect osteoclastogenesis.

In the bone tissues of Col2.3-ECFP/TRAP-tdTomato mice, in which mOBs and mOCs were labeled with ECFP and tdTomato, respectively^19^, intravital imaging revealed that TRAP-positive mOCs were preferentially present in close vicinity of ECFP-positive mOBs actively secreting SOVs (Fig. 3d). Quantitative microscopic analysis showed that TRAP-positive mOCs coincided with mOBs surrounded by SOVs (Fig. 3e). These results suggest that SOV-treated osteoblasts stimulate osteoclast differentiation.

Because SOVs inhibited osteoblast differentiation and SOV-treated osteoblasts promoted osteoclastogenesis, we evaluated the effects of SOVs on bone formation in vivo. We established bilateral calvarial defects in wild-type (WT) mice. Bone defects were exposed to hydrogels impregnated with PBS or total SOVs for 8 weeks (Fig. 3f). The affected areas were significantly larger in the SOV-treated group than in the PBS-treated group (Fig. 3g, h), indicating that SOVs inhibited bone repair in vivo.

### SOV-derived miR-143-3p suppress osteoblast differentiation by targeting *Cbfb* mRNA

**Fig. 4:**
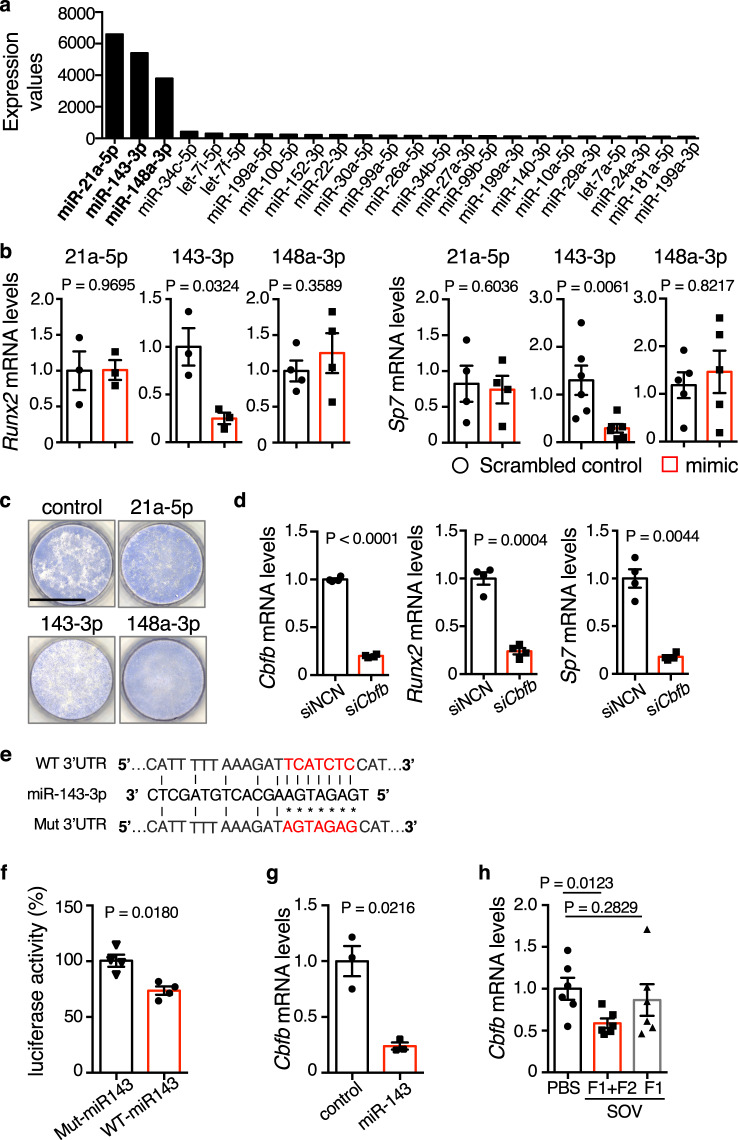
SOV-derived miR-143-3p inhibits osteoblast differentiation by targeting *Cbfb* mRNA. **a** Next-generation sequencing-mediated miRNA profiling of SOVs. Top-ranked expression values of miRNAs in SOVs from primary osteoblasts of 10 Col2.3-ECFP mice. **b** *Runx2* and *Sp7* mRNA levels in MC3T3-E1 cells transfected with scrambled, miR-21a-5p, miR-143-3p, or miR-148a-3p mimics (The *Runx2* expression in miR-21a-5p and miR-143-3p groups: *n* = 3; the *Runx2* expression in a miR-148a-3p group and the *Sp7* expression in a miR-21a-5p group: *n* = 4; the *Sp7* expression in a miR-148a-3p group: *n* = 5; the *Sp7* expression in a miR-143-3p group: *n* = 6 independent experiments, respectively). **c** Representative images of ALP activity in MC3T3-E1 cells transfected with scrambled, miR-21a-5p, miR-143-3p, or miR-148a-3p mimics. Scale bar: 10 mm. **d** *Cbfb*, *Runx2*, and *Sp7* mRNA levels in MC3T3-E1 cells transfected with siRNA negative control or *Cbfb* siRNA. siNCN: siRNA negative control; si*Cbfb*: *Cbfb* siRNA (*n* = 4 independent experiments). The exact *p* value of the *Cbfb* expressioin: 2.34811^−9^. **e** *Cbfb* 3′ UTR constructs cloned into luciferase reporter vectors. WT 3′ UTR: wild-type sequence of the *Cbfb* 3′ UTR; miR-143-3p: miR-143-3p sequence; Mut 3′ UTR: mutant sequence of the *Cbfb* 3′ UTR. **f** Percentages of luciferase activity. Mut-miR143: mutated *Cbfb* construct with miR-143-3p mimics; WT-miR143: WT *Cbfb* construct with miR-143-3p mimics (*n* = 4 independent experiments). **g** *Cbfb* mRNA levels in MC3T3-E1 cells treated with scrambled control or miR-143-3p mimics. Control: scrambled controls; miR-143: miR-143-3p mimics (*n* = 3 independent experiments). **h** *Cbfb* mRNA levels in primary osteoblasts treated with PBS, total SOVs (F1 + F2), or SOV-F1 (*n* = 6 biological replicates per group). Data are means ± SEMs. Statistical significance was determined by two-tailed paired *t*-test in (**b**, **d**, **f**, **g**) and by one-way ANOVA with Dunnett’s multiple comparison post hoc test in (**h**).

To identify the mechanism by which SOVs suppress osteoblast differentiation, we analyzed miRNAs commonly found in SOVs^21,22^ via next-generation sequencing. We identified miR-21a-5p, miR-143-3p, and miR-148a-3p as miRNAs enriched in SOVs (Fig. 4a). To determine the roles of these miRNAs in osteoblast differentiation, we transfected MC3T3-E1 cells with miRNA mimics and analyzed the expression levels of the osteoblastic genes *Runx2* and *Sp7*. The miR-143-3p mimics significantly suppressed the expression of *Runx2* and *Sp7* (Fig. 4b); they also inhibited ALP activity (Fig. 4c). The effects of miR-143-3p mimics on osteoblast differentiation were similar to the effects of total SOVs. Furthermore, transfection of miR-143-3p mimics significantly increased the expression of *Rankl* and suppressed the expression of *Opg* in MC3T3-E1 cells, consistent with the effects of SOV-treated osteoblasts on osteoclastogenesis (Supplementary Fig. S4a, b).

To determine the molecular mechanisms by which miR-143-3p controls the expression levels of *Runx2*, *Sp7*, and *Rankl*, we analyzed its potential targets using Target Scan 7.2 and miRbase 22.1. Among hundreds of predicted mRNA targets, we focused on core-binding factor β (*Cbfb*), which may regulate the expression levels of *Runx2* and *Sp7*. Cbfb has been reported to form heterodimers with Runx proteins, thereby enhancing their DNA-binding capacity^30^. Osteoblast-specific *Cbfb* conditional deletion in *Col1a1*-cre mice has also been reported to downregulate *Runx2* and *Sp7*, while inhibiting osteoblast differentiation^31–33^. Consistent with previous findings, *Cbfb* silencing in MC3T3-E1 cells significantly decreased the *Runx2* and *Sp7* mRNA levels (Fig. 4d). To assess the ability of miR-143-3p to directly bind to *Cbfb* mRNA, we generated luciferase reporter constructs containing WT or mutant *Cbfb* mRNA binding sites (Fig. 4e). Each construct was transfected into MC3T3-E1 cells in combination with miR-143-3p mimics. The miR-143-3p mimics significantly inhibited luciferase activity in MC3T3-E1 cells expressing the WT *Cbfb* binding site, but they did not inhibit luciferase activity in cells expressing the mutant binding site (Fig. 4f). Furthermore, the expression level of *Cbfb* was significantly decreased in MC3T3-E1 cells transfected with miR-143-3p mimics (Fig. 4g). These findings suggest that miR-143-3p binds directly to *Cbfb* mRNA. miR-143-3p drives osteoblast differentiation by targeting histone deacetylase 7 (*Hdac7*)^34^. Therefore, we performed a luciferase assay to evaluate the ability of miR-143-3p to bind to *Hdac7* mRNA. miR-143-3p mimics did not affect luciferase activity in MC3T3-E1 cells expressing WT or mutant *Hdac7* binding sites (Supplementary Fig. S4c, d). Total SOVs (F1 + F2), but not SOV-F1, significantly decreased the mRNA level of *Cbfb* in primary osteoblasts (Fig. 4h). In contrast, neither total SOVs nor SOV-F1 affected *Hdac7* expression (Supplementary Fig. S4e), indicating that miR143 in SOVs does not affect the expression level of *Hdac7* in SOV-treated osteoblasts. Collectively, these findings suggest that SOV-derived miR-143-3p suppresses osteoblast differentiation by targeting *Cbfb*.

To confirm the transfer of miR-143-3p enclosed in SOVs to recipient osteoblasts, we visualized miR-143-3p in SOVs. We labeled endogenous miR-143-3p by transfecting Alexa555-conjugated miR-143-3p inhibitor into EGFP-expressing MC3T3-E1 cells and collected SOVs from the transfected cells. These SOVs were administered to non-labeled MC3T3-E1 cells and observed by confocal microscopy. Alexa555-labeled miR-143-3p colocalized with EGFP-positive vesicles in non-labeled MC3T3-E1 cells (Supplementary Fig. S4f). This finding suggests that miR-143-3p is enclosed in SOVs and transferred into recipient cells.

### miR-143/145 conditional knockout increases bone mass and promotes bone mineralization in mice

**Fig. 5:**
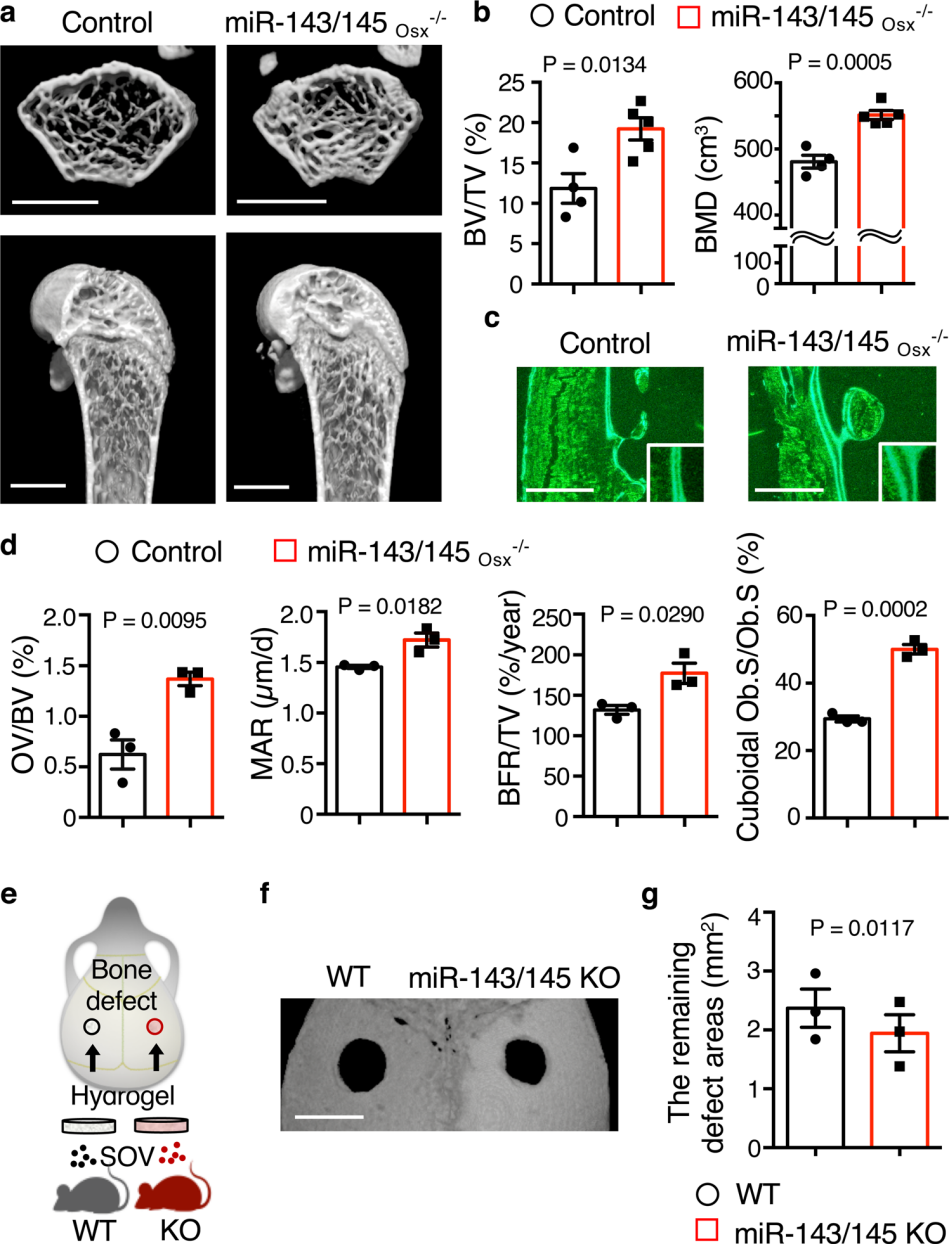
miR-143/145 conditional KO increases bone mass and promotes mineralization in mice. **a** Representative micro-CT images of the femurs of 9-week-old control (*n* = 4 biological replicates) and osteoblast-specific miR-143/145 KO (miR-143/145_Osx_^−/−^) mice (*n* = 5 biological replicates). Scale bars: 1000 μm. **b** Micro-CT-based assessment of metaphyseal regions of distal femurs of 9-week-old control (*n* = 4 biological replicates) and miR-143/145_Osx_^−/−^ (*n* = 5 biological replicates) mice. BV: bone volume; TV: tissue volume; BMD: bone mineral density. **c**, **d** Bone morphometric analysis of metaphyseal regions of distal femurs of 9-week-old control and miR-143/145_Osx_^−/−^ mice (*n* = 3 biological replicates per group). Calcein double signals in a representative femur section from a control or miR-143/145_Osx_^−/−^ mouse on cortical surfaces and magnified view (inset). Scale bars: 100 µm (**c**). Bone formation parameters (**d**). OV: osteoid volume; MAR: mineral apposition rate; BFR: bone formation rate; Ob.S: osteoblast surface. **e** Transplantation model of calvarial defects. Artificial bilateral defects on mouse calvaria were treated with total SOVs from miR-143/145 KO or WT mice. **f** Representative micro-CT image of calvarial defects at 2 weeks after treatment with SOVs from miR-143/145 KO or WT mice (*n* = 3 biological replicates per group). Scale bar: 2000 µm. **g** Remaining defect areas. miR-143/145 KO: mice treated with total SOVs from miR-143/145 KO mice; WT: mice treated with SOVs from WT mice (*n* = 3 biological replicates per group). Data are means ± SEMs. Statistical significance was determined by two-tailed unpaired *t*-test in (**b**, **d**) or by two-tailed paired *t*-test in (**g**).

To further investigate the role of miR-143-3p in bone metabolism in vivo, we generated an osteoblast-specific miR-143/145 knockout (KO) mouse model (miR-143/145_Osx_^−/−^) by crossing miR-143/145 ^flox/flox^ mice with Osx1-Cre mice, in which Cre expression is regulated by the *Osterix* promoter. Because miR-143 and miR-145 are on the same gene locus, the miR-143 and -145 genes were floxed in these mice. Osx-expressing cells during the perinatal period give rise to osteoblasts, stromal cells, adipocytes, and perivascular cells; in the postnatal period, the expression of *Osterix* is restricted to osteoblasts^35^. In Osx1-Cre mice, Cre-mediated recombination was under the control of doxycycline. Using this system, we generated miR-143/145_Osx_^−/−^ mice expressing Cre exclusively at the postnatal period when mated to floxed-transgenic strains. Compared with control 9-week-old mice, miR-143/145_Osx_^−/−^ mice expressed low levels of miR-143-3p in primary osteoblasts (Supplementary Fig. S5a). The mRNA levels of *Runx2*, *Sp7*, and *Bglap* in primary osteoblasts were significantly higher in miR-143/145_Osx_^−/−^ mice than in littermate control mice (Supplementary Fig. S5b). Micro-computed tomography (CT) analyses revealed that, compared with control mice, miR-143/145_Osx_^−/−^ mice had an increased bone volume/tissue volume (BV/TV) and bone mineral density (BMD) (Fig. 5a, b). Similarly, bone morphometric analyses showed that the parameters of bone formation—including osteoid volume/bone volume (OV/BV), mineral apposition rate (MAR), and bone formation rate/tissue volume (BFR/TV)—were significantly increased in miR-143/145_Osx_^−/−^ mice, compared with littermate control mice (Fig. 5c, d). Although the osteoblast surface/bone surface (Ob.S/BS) was comparable between miR-143/145_Osx_^−/−^ and control mice (Supplementary Fig. S5c), the cuboidal Ob.S/Ob.S (ratio of Ob.S of mOBs to Ob.S of total osteoblasts) was significantly higher in miR-143/145_Osx_^−/−^ mice than in control mice (Fig. 5d). Therefore, osteoblast-specific miR-143/145 depletion increased bone mass and promoted mineralization.

### miR-143/145 conditional knockout decreases osteoclast activity after parathyroid hormone administration in vivo

Because deletion of miR143-3p in osteoblasts in vivo using miR-143/145_Osx_^−/−^ mice did not influence any parameters of osteoclast formation or function (Supplementary Fig. S5c), we performed bone morphometric analyses of miR-143/145_Osx_^−/−^ mice under the high bone-metabolic turnover condition. We intermittently injected parathyroid hormone (PTH) into WT and miR-143/145_Osx_^−/−^ mice, then performed micro-CT analysis, bone morphometric analysis, and measurement of the urine C-terminal telopeptide-1 (CTX-1) level, which reflects osteoclast activity^36^. Micro-CT and bone morphometric analyses revealed that there were no significant differences in BV/TV between PTH-treated WT mice and PTH-treated miR-143/145_Osx_^−/−^ mice (Supplementary Fig. S6a, c). The urine CTX-1 level (corrected for urine creatinine) was significantly lower in miR-143/145_Osx_^−/−^ mice than in WT mice (Supplementary Fig. S6b). Consistent with those findings, morphometric analyses revealed that eroded surface/bone surface (ES/BS), osteoclast number/bone surface (N.Oc/BS), and osteoclast surface/bone surface (Oc.S/BS) were significantly lower in PTH-treated miR-143/145_Osx_^−/−^ mice than in PTH-treated WT mice (Supplementary Fig. S6c). Therefore, miR-143/145 osteoblast-specific knockout mice showed significantly decreased osteoclast activity and number compared with WT mice, indicating that miR-143/145 in osteoblasts promotes osteoclastogenesis in vivo under a high bone-metabolic turnover condition (Supplementary Fig. S6b, c).

### MiR-143/145-deleted SOVs reversed the inhibition by SOVs of bone repair

To investigate the effects of miR-143 in SOVs on osteoblast differentiation and osteoclastogenesis, we analyzed the effects of SOVs from miR-143/145_Osx_^−/−^ mice on *Runx2*, *Sp7*, and *Rankl* expression levels in osteoblasts in vitro. The miR-143/145-deleted SOVs significantly reversed the inhibition (by WT SOVs) of *Runx2* and *Sp7* expression (Supplementary Fig. S7a). In addition, miR-143/145-deleted SOVs significantly reversed the enhancing effect of WT SOVs on *Rankl* expression in osteoblasts (Supplementary Fig. S7b).

Next, to analyze the function of miR-143 in SOV on bone formation in vivo, bilateral calvarial defects in WT mice were exposed to gelatin hydrogels impregnated with total SOVs from miR-143/145 KO or WT mice (Fig. 5e). At 2 weeks after the procedure, the affected areas were significantly smaller in areas treated with total SOVs from miR-143/145 KO mice, than in areas treated with total SOVs from WT mice (Fig. 5f, g), suggesting that miR-143 in SOVs impairs bone repair.

**Fig. 6:**
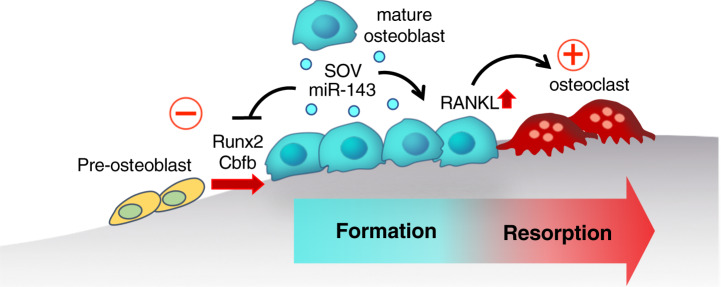
Graphical abstract of the role of small osteoblast vesicles in bone remodeling. Interactions among mOBs via SOVs inhibit osteoblastic bone formation and stimulate osteoclast formation, thus switching from the bone formation phase to the bone resorption phase.

In summary, these results suggest that SOVs inhibit osteoblastic bone formation and stimulate osteoclast formation through an miR-143-mediated mechanism, which promotes reciprocal reserve phase switch (Fig. 6).

## Discussion

Bone formation by osteoblasts is a long-lasting phase that may last several months and is tightly regulated during bone remodeling^37^. Although the signaling and transcriptional regulation of osteoblast differentiation have been investigated^38,39^, the terminating factors of bone formation and initiating factors of subsequent bone resorption remain elusive. Using an intravital bone imaging approach, we identified intercellular communication among osteoblasts via SOVs and demonstrated negative regulation of bone formation and positive regulation of bone resorption by SOVs derived from mOBs.

Osteoblasts co-cultured with SOVs inhibited osteoblastogenesis and enhanced the osteoclastogenesis of BMMs. The effect of SOVs on osteoclastogenesis was not a direct effect of SOVs on BMMs; it was caused by phenotypic changes, such as the upregulation of *Rankl* in SOV-treated osteoblasts. Consistent with this finding, mOCs were exclusively induced on nodules that had been created by total SOV-treated osteoblasts in the co-culture system, indicating that osteoblast–osteoblast communication via SOVs is important for spatiotemporal regulation of osteoclastogenesis. These observations suggest that communication among osteoblasts by means of SOVs induces a phase transition from bone formation to bone resorption as a spatiotemporal bone remodeling regulation mechanism.

It is widely accepted that bone formation follows bone resorption^2^; however, our findings suggest that osteoblast–osteoblast communication via SOVs can directly induce osteoclastogenesis, providing further insights into bone homeostasis. The possibility of immediate transition to bone resorption after the bone formation phase without a resting phase remains controversial^6^. We believe that direct transition from bone formation to bone resorption may happen under high turnover conditions and dysregulated remodeling conditions, such as osteoporosis^40,41^ and bone repair. Future studies are needed to confirm this hypothesis.

We identified *Cbfb* mRNA as a target of miR-143-3p in osteoblasts. The bone phenotype of miR-143/145_Osx_^−/−^ mice was consistent with the phenotype of *Cbfb* conditional KO (*Cbfb*_col1a1_^−/−^) mice^33^. *Cbfb*_col1a1_^−/−^ mice in the postnatal period exhibited reduced bone mass, MAR, and *Runx2* expression. Although the number of osteoblasts was unchanged, the number of active osteoblasts decreased in *Cbfb*_col1a1_^−/−^ mice^33^. Notably, miR-143/145_Osx_^−/−^ mice exhibited a phenotype opposite to the findings in *Cbfb*_col1a1_^−/−^ mice (i.e., increased bone mass, MAR, *Runx2* mRNA expression, and ratio of mOBs to total osteoblast area [cuboidal Ob.S/Ob.S]). MiR-143 was previously shown to inhibit *Sp7* mRNA expression in osteoblasts, as indicated by luciferase assay results using human *Sp7* mRNA sequences^42,43^. However, according to two major databases, Target Scan 7.2 and miRBase 22.1, murine *Sp7* mRNA does not have complementary base sequences to bind to mmu-miR-143-3p. Thus, the suppressed *Sp7* expression in murine osteoblasts transfected with miR-143-3p mimics was presumably not caused by direct binding of miR-143-3p mimics to *Sp7* mRNA; it was likely dependent on the decreased expression of *Runx2* caused by miR-143-3p binding to *Cbfb* mRNA.

In addition to the effect of miR-143-3p on osteoblast differentiation, we demonstrated that miR-143-3p increased the *Rankl/Opg* ratio in MC3T3-E1 cells. Although increasing *Rankl/Opg* in osteoblasts should enhance osteoclastogenesis, the parameters of osteoclast formation and function were not affected in miR-143/145_Osx_^−/−^ mice at steady state (Supplementary Fig. S5c), consistent with the phenotype of *Cbfb*_col1a1_^−/−^ mice^33^. However, the parameters were significantly decreased in miR-143/145_Osx_^−/−^, compared with control mice, following intermittent PTH administration. The phenotypes of osteoclast formation and function in miR-143/145_Osx_^−/−^ mice were enhanced by intermittent PTH administration with increasing bone turnover. In addition to endogenous effects in osteoblasts, we evaluated the effects of miR-143 in SOVs. MiR-143-containing SOVs significantly upregulated *Rankl* expression in SOV-treated osteoblasts; this change was reversed by miR-143/145-deleted SOVs. This finding suggests that miR-143-3p in SOVs induced a phenotypic change of osteoblasts to enhance osteoclastogenesis. Therefore, miR-143 is essential for the ability of SOVs to inhibit bone formation and promote bone resorption.

Although miR-143 directly binds to *Cbfb* mRNA and inhibits osteoblast differentiation, the mechanism by which miR-143 increases *Rankl* expression in osteoblasts is unknown. Runx2 regulates *Rankl* expression by condensing the chromatin structure at the *Rankl* enhancer and promoter regions in a histone deacetylase 3-dependent manner^44^. Decreased *Runx2* expression might have led to *Rankl* upregulation in osteoblasts treated with SOVs or miR-143-3p mimics. However, the miR-143/145-deleted SOVs did not totally cancel the effect of WT SOVs on *Runx2* and *Rankl* expression, implicating other factors in SOVs. Other proteins and nucleic acids in SOVs may also affect osteoblast differentiation and function. Future studies are needed to identify functional proteins and/or other components in these vesicles.

In conclusion, our findings provide an insight into the in vivo interactions among mOBs via SOVs and demonstrate that SOVs are critical factors regulating reciprocal reversal switch in a miR-143-dependent mechanism.

## Methods

### Mouse model

C57BL/6J mice were obtained from CLEA Japan (Tokyo, Japan). The generation of Col1a1*2.3-ECFP and TRAP-tdTomato transgenic mice was described previously^18,19^. miR-143/145-floxed mice and miR-143/145 KO mice^45^ were kindly provided by Thomas Boettger and Thomas Braun, Max-Plank-Institute, Germany. To generate the loxP-mice, we generated a miR-143/145 lox/lox targeting vector by subcloning the miR-143/145 locus^45^. A loxP site was inserted into the HindIII site 1 kb upstream of miR-143, and a second loxP site plus a neomycin resistance cassette was inserted into the HindIII site 2.3 kb downstream of miR-145. Mice were generated as described previously^45^. Osx1-GFP:Cre mice were obtained from the Jackson Laboratory (Stock Number: 006361).

miR-143/145_Osx_^−/−^ mice and their littermates were fed a diet containing doxycycline 0.02% (v/v) via breastfeeding until becoming 4 weeks old. Nine-week-old mice were analyzed for bone phenotypic changes. Male mice (10–20 weeks of age) were used unless indicated otherwise. The number of animals used is indicated in the corresponding figure legend. All mice were housed in specific pathogen-free facilities under a 12 h light/dark cycle and controlled temperature (19–23 °C) and humidity (55 ± 10%). Mice were given standard laboratory chow diet and water *ad libitum* unless indicated otherwise. Animal studies were approved by the Institutional Review Board of Osaka University.

### Cell culture

In vitro osteoblast differentiation has been described previously^19,46^. Briefly, bone marrow cells (5.3 × 10^5^ per cm^2^) were collected by flushing long bones of mice and maintained for 3 days in α-minimum essential medium (α-MEM) containing 10% fetal bovine serum (FBS) and 1% penicillin–streptomycin at 37 °C in a humidified incubator containing 5% CO_2_. The cells were maintained in osteogenic medium (α-MEM, 10% FBS, 1% penicillin–streptomycin, 50 mg/mL ascorbic acid, and 10 mM beta-glycerophosphate) for 14 days to promote osteoblastic differentiation and mineralization. The osteogenic medium was refreshed every 3 days.

MC3T3-E1, a clonal osteoblastic cell line derived from newborn mouse calvaria, was purchased from the American Type Culture Collection (CRL-2593™: ATCC, Manassas, VA, USA). Cells (1.05 × 10^4^ per cm^2^) were maintained for 2 days in α-MEM containing 10% FBS and 1% penicillin–streptomycin. For osteoblastic differentiation and mineralization, cells were maintained in osteogenic medium, which was refreshed every 3 days.

To generate BMMs, non-adherent bone marrow cells from C57BL/6J mice were seeded in a 10 cm Petri dish and cultured in α-MEM containing 10% FBS, 1% penicillin–streptomycin, and 10 ng/mL M-CSF. After 3 days, adherent cells were collected in enzyme-free cell dissociation buffer (Millipore, Burlington, MA, USA) at 37 °C and used as BMMs.

### Intravital multiphoton imaging

Col1a1*2.3-ECFP mice and Col2.3-ECFP/TRAP-tdTomato double transgenic reporter mice were anesthetized with isoflurane (Escain; 2.5% vaporized in an 80:20 mixture of O_2_ and air), and the hair on the neck and the scalp was removed using hair removal lotion (Epilat). The front-parietal skull was exposed after the skin incision, and the head was immobilized in a custom-made stereotactic holder^19^. Intravital multiphoton imaging was performed as described previously^15–19^. The imaging system for Col1a1*2.3-ECFP mice was composed of a multiphoton microscope (LSM 780 NLO; Carl Zeiss) driven by a laser (Chameleon Vision II Ti: Sapphire; Coherent) at 860 nm and an upright microscope equipped with a 20× water immersion objective (W Plan-APOCHROMAT; Carl Zeiss). The microscope was enclosed in an optimally customized chamber in which the temperature was controlled to warm the anesthetized mice. The heart rate of the mice was monitored by electrocardiography. Fluorescent cells were detected by a spectral imaging microscope. The step size was adjusted to 2 μm to create Z-stack images; five slices were captured as the depth was 10 µm. Images were recorded with pixel dimensions of 0.26 μm and a time interval of 5 min. Raw imaging data were processed to 3D images and maximum intensity projection (MIP) on Z with NIS Elements software (Nikon). Constant g corrections were applied to all images (g = 0.65) with NIS Elements software to enhance the signal-to-noise ratio. MIP data were edited to surface images with Imaris software (Bitplane). The tracking analysis was performed using the surface function. Surface objects were created manually and filtered according to tracking time (>20 min).

The imaging system for Col2.3-ECFP/TRAP-tdTomato mice consisted of an upright multiphoton microscope (A1R MP+; Nikon) with a 25× water immersion objective (CFI75 Apo 25XC W 1300; Nikon). The system was driven by lasers (Chameleon Vision II Ti: Sapphire; Coherent, Inc.); the main laser was tuned to 860 nm to detect ECFP and second harmonic generation, and the sub-laser was tuned to 1040 nm to detect tdTomato. Multi-fluorescence images were acquired under a Nikon upright microscope using four external non-descanned detectors equipped with dichroic and emission filters, including an infrared-cut filter (DM685), three dichroic mirrors (DM458, DM506, and DM605), and three emission filters (417/60 for the second harmonic generation image, 480/40 for ECFP, 583/22 for tdTomato).

### In vitro time-lapse imaging

Primary osteoblasts from Col2.3-ECFP mice cultured for 10–14 days and CD63-GFP MC3T3-E1 cells cultured for 3 days were observed, and time-lapse images were captured using a Nikon A1-Si confocal laser scanning microscope. Primary osteoblasts and CD63-GFP MC3T3-E1 cells were excited at 457 nm and 561 nm for primary osteoblasts and 488 nm for CD63-GFP MC3T3-E1 cells (Coherent). Autofluorescence was collected with photomultiplier-type detectors at wavelength emission windows of 482/35 nm for the 457 nm laser to detect ECFP, 525/50 nm for the 488 nm laser to detect EGFP, and 595/50 nm for the 561 nm laser to detect PKH26. Images were recorded with pixel dimensions of 0.31 or 0.63 µm. Raw imaging data were edited with NIS Elements software (Nikon). Constant g corrections were applied to all images (g = 0.65) to enhance the signal-to-noise ratio. MIP data were edited to surface images with Imaris software (Bitplane).

### Isolation of SOVs

SOVs were isolated from primary osteoblasts or MC3T3-E1 cells according to a previously described procedure^47^. Briefly, the culture medium was replaced with EV-free osteogenic medium (containing 10% EV-free FBS prepared by centrifugation at 70,000 × *g* overnight instead of 10% FBS) 2 days before SOV collection. The culture medium was collected, and cells were treated with 0.2% collagenase P (Roche) for 1 h at 37 °C followed by 0.25% trypsin (containing 0.02% EDTA) for 10 min at 37 °C. The culture medium and digested cells were centrifuged at 300 × *g* for 5 min at 4 °C to separate cells, and the supernatant was centrifuged at 2000 × *g* for 20 min at 4 °C to remove debris. The supernatant was further centrifuged at 16,500 × *g* for 20 min at 4 °C. The pellet containing SOVs was resuspended in PBS and divided into two groups: total SOVs (F1 + F2) and SOV-F1 (0.22-µm filtration). Both groups were treated with 0.5 mg/mL RNase A (Nippon Gene) at 37 °C for 20 min and were centrifuged at 16,500 × *g* for 20 min to wash. For proteinase K treatment, SOVs were treated with proteinase K (final concentration: 0.1 mg/mL) for 20 min at 37 °C, then incubated for 5 min at 90 °C to inactivate proteinase K. EV experiments were performed based on the criteria presented by the International Society of Extracellular Vesicles position paper in 2018^26^.

### PKH-labeling of SOVs

SOVs were collected from primary osteoblasts cultured for 14 days. SOVs were then labeled with PKH26 Red Fluorescent Cell Linker Kit (Sigma-Aldrich) at room temperature for 5 min, blocked with FBS, and washed four times with PBS in accordance with the manufacturer’s instructions. Primary osteoblasts cultured for 10–14 days were treated with PKH-labeled SOVs and were observed under a confocal microscope.

### Generation of stable cell lines

The lentiviral vector pCT-CD63-GFP (System Biosciences) and a packaging plasmid for generation of a CD-63-GFP MC3T3-E1 cell line or the pMYs-IRES-GFP Retroviral Vector (CELL BIOLABS) for generation of a MC3T3-E1 GFP cell line were transfected into HEK293T cells using PEI max (Polysciences). MC3T3-E1 cells were transduced with virus-containing medium from transfected HEK293T cells and polybrene (Merck) 48 h after transfection. Transduced MC3T3-E1 cells expressing GFP were selected using a cell sorter (SONY), and single cells were cultured to establish a cell line.

### Imaging flow cytometry

Primary osteoblast samples from Col1a1*2.3-ECFP transgenic mice were prepared after 14 days of culture and were treated with PKH-labeled total SOVs. Samples were then analyzed using an ImageStreamX Mark II cytometer (Amnis, EMD Millipore) at a low flow rate and high sensitivity using 60-fold magnification. Cells were collected by centrifugation at 300 × *g* after treatment with 0.2% collagenase P (Roche) for 1 h at 37 °C, followed by 0.25% trypsin (containing 0.02% EDTA) for 10 min. Single-cell suspensions (2 × 10^7^/mL) in flow cytometry (FACS) buffer were incubated with PE-Cy7 conjugated anti-mouse CD45 antibody (BioLegend) diluted in FACS buffer at 1:100 ratio for 15 min. CFP, CD45-PE-Cy7, and PKH26 were excited at 495 nm (CFP) and 488 nm (CD45-PECy7 and PKH26) and were detected at 435–505 nm (CFP), 745–785 nm (CD45-PECy7), and 642–745 nm (PKH26). Flow cytometric data were analyzed using IDEAS software (Amnis, EMD Millipore).

### Transmission electron microscopy

Collected total SOVs (F1 + F2) or SOV-F1 were fixed with iPGell (Geno Staff). Samples were washed and post-fixed in 1% osmium tetroxide at 4 °C for 90 min. Subsequently, samples were dehydrated in graded ethanol with propylene oxide and embedded in epoxy resin. Semi-thin sections (75 nm) were mounted on copper grids and observed using an H-7650 transmission electron microscope (Hitachi Electronic Instruments).

### Nanoparticle tracking analysis

Nanoparticle tracking analysis was performed using Nanosight LM10 and NTA2.3 software (NanoSight). Samples were diluted 100-fold with PBS, and 30-s videos were recorded at the same camera level and detection threshold on the same day. The temperature was monitored throughout the measurements.

### Treatment of SOVs

Primary osteoblasts cultured in 24-well plates for 6 days were treated with PBS, total SOVs (F1 + F2), or SOV-F1. SOVs were collected from primary osteoblasts that had been cultured for 14 days. The final concentrations of total SOVs (F1 + F2) and SOV-F1 were 1–5 × 10^9^/mL. MC3T3-E1 cells cultured in 24-well plates for 21 days were treated with PBS, total SOVs (F1 + F2), or SOV-F1. SOVs were collected from MC3T3-E1 cells that had been cultured for 28 days. The final concentrations of total SOVs (F1 + F2) and SOV-F1 were 1–5 × 10^9^/mL. Total RNAs were extracted 2 days after treatment, and ALP staining was performed 5 days after treatment. For collagen and Alizarin Red S staining, we treated primary osteoblasts cultured in 24-well plates for 3 days with PBS, total SOVs (F1 + F2), or SOV-F1 from primary osteoblasts that had been cultured for 14 days, then stained them 3 days after treatment.

### RNA isolation and quantitative real-time PCR

Total RNA and cDNA were prepared using the Maxwell 16 LEV RNA purification kit (Promega) and Superscript III reverse transcriptase (Thermo Fisher Scientific) in accordance with the manufacturer’s instructions. Quantitative real-time PCR (qPCR) was performed using a TP800 Dice Real-Time Thermal Cycler System (TaKaRa). Gene expression was calculated relative to that of the housekeeping gene β-actin. The primer sequences used are listed in Supplementary Table 1.

### ALP and TRAP staining

ALP substrate solution was prepared by dissolving 0.006% (w/v) Naphthol AS-MX phosphate (Sigma-Aldrich) in 0.1 M Tris (pH 8.0) and adding 0.1% (w/v) Fast blue BB salt (Sigma-Aldrich). After fixation in 4% paraformaldehyde, cells were stained with ALP substrate solution for 10 min at room temperature in the dark and were rinsed with distilled water. For quantitative analyses, ALP activities were assessed using a TRACP & ALP Assay Kit (TaKaRa Bio) in accordance with the manufacturer’s instructions. Cells were washed with saline and were lysed using a commercial lysis solution. Samples were treated with ALP substrate for 15 min at 37 °C, and the absorbance at 405 nm was measured using a microplate reader.

TRAP substrate solution was prepared by dissolving 0.01% (w/v) Naphthol AS-MX phosphate (Sigma-Aldrich) in TRAP buffer (pH 5.0, 66 mM sodium acetate and 45 mM sodium tartrate) supplemented with 0.06% (w/v) Fast Red Violet LB salt (Sigma-Aldrich). After fixation in 4% paraformaldehyde, cells were permeabilized with acetone ethanol (1:1) solution and stained with TRAP substrate solution for 5 min at room temperature.

### Collagen staining and Alizarin Red S staining

For collagen staining, after fixation in Kahle Fixative, cells were stained with a dye solution in accordance with the manufacturer’s instructions (Chondex). For Alizarin Red S staining, after fixation in 4% paraformaldehyde cells were stained with 2% Alizarin Red S solution (Fujifilm Wako Pure Chemical Corporation).

### Cell death analysis

Primary osteoblasts cultured for 6 days were treated with PBS, total SOVs (F1 + F2), or SOV-F1 for 6 days. Cells were analyzed using a cell sorter (SH800, Sony) to detect 7-AAD signals. Single-cell suspensions (1 × 10^6^/mL) were incubated with CD45-PECy7 (BioLegend) diluted in FACS buffer at 1:100 ratio for 15 min, and stained with 10 μg/ml 7-AAD (BD Biosciences) immediately before analysis to detect dead cells. Flow cytometry data were analyzed using FlowJo software (TreeStar).

### Co-culture system for osteoclastogenesis in vitro

BMMs were co-cultured with total SOV-treated osteoblasts or PBS-treated osteoblasts using a modified osteoclast culture method^48^. Primary osteoblasts cultured for 6 days were treated with total SOVs or PBS. The culture medium containing SOVs or PBS was removed 2 days after treatment; BMMs (0.63 × 10^5^/cm^2^) were co-cultured with the osteoblasts in osteogenic medium with 100 ng/mL 1 alpha,25-dihydroxyvitamin D3 (Calcitriol, Fujifilm Wako Pure Chemical Corporation). Three days later, TRAP-positive multinucleated cells were counted as mOCs after TRAP staining and qRT-PCR of mature osteoclast marker genes were performed.

To evaluate the direct effect of SOVs on osteoclastogenesis, BMMs (1.5 × 10^5^/cm^2^) were cultured for 2 days with 10 ng/mL M-CSF. SOVs (final concentration of 1–5 × 10^9^/mL) were added to the BMMs with or without 10 ng/mL M-CSF. Three and seven days later, TRAP staining was performed.

### miRNA isolation and qPCR

Total RNA containing miRNAs was prepared using a miRNeasy Mini Kit (Qiagen). qPCR was performed using a TaqMan Advanced miRNA kit in accordance with the manufacturer’s instructions. The expression level of each miRNA was calculated relative to that of miR-423-5p.

### Next-generation miRNA sequencing

SOV-derived RNAs from primary osteoblasts cultured for 14 days were isolated using miRNeasy Mini Kit (Qiagen). Small RNA libraries were constructed using the NEBNext Small RNA Library Prep Set for Illumina (NEB) in accordance with the manufacturer’s instructions, and libraries were sequenced on the HiSeq 2500 platform (Illumina) in 75-base pair single-end reads. The miRNA-seq analysis was conducted using CLC Genomics Workbench v9.5.3 (Qiagen) in accordance with the small RNA alignment and small RNA analysis pipeline using default parameters. Adapters were trimmed to retain only reads of length 15–25 bp. Of 1,927,614 trimmed reads, 832,524 were then mapped and annotated against the miRBase (version 21). Read counts of the annotated miRNAs were exported from the CLC Genomics Workbench. Raw data were deposited in the NCBI GEO database (GSE144512).

### Transfection of miRNA mimics

MC3T3-E1 cells were maintained with serum- and antibiotic-free medium for 24 h. miRNA mimics of miR-143-3p, miR-21-5p, and miR-148-3p and mimic negative control (mirVana™ miRNA mimics, Thermo Fisher Scientific) were transfected into MC3T3-E1 cells using Lipofectamine RNAiMAX (Thermo Fisher Scientific). RNA was extracted 2 days after transfection.

### Transfection of siRNAs

Cbfb siRNAs (Stealth RNAi™ siRNA, Thermo Fisher Scientific) or negative control siRNAs (Stealth RNAi™ siRNA Negative Control Lo GC, Thermo Fisher Scientific) were transfected into MC3T3-E1 cells using Lipofectamine RNAiMAX (Thermo Fisher Scientific). RNA was extracted 2 days after transfection.

### Luciferase reporter assay

WT *Cbfb* or *Hdac7* sequence fragments containing predicted miR-143-3p binding sites were chemically synthesized and cloned downstream of the luciferase gene in the pmirGLO Dual-Luciferase miRNA Target Expression Vector (Promega) between *SacI* and *XbaI* sites. Fragments with mutant binding sites were also synthesized and cloned as controls. MC3T3-E1 cells cultured in 96-well plates (1 × 10^4^/well) were co-transfected with the recombinant reporter plasmids and miR-143-3p mimic or scramble negative controls using Lipofectamine 2000 (Invitrogen). Cells were harvested 48 h after transfection, and luciferase activity was measured using the Dual-Glo Luciferase Assay System (Promega) and Luminescent Micro Plate Reader (Centro XS3 LB960, Berthold Technologies). Firefly luciferase activity was normalized to that of Renilla luciferase.

### Visualization of transfer of miR-143-3p in SOVs

MC3T3-E1 EGFP cells were transfected with Alexa Fluor 555-conjugated miR-143-3p inhibitor (mirVana miRNA Inhibitor, Thermo Fisher Scientific). SOVs were collected from the transfected cells 1 day after transfection. MC3T3-E1 cells were treated with the collected SOVs, and the cells were observed with a confocal microscope (Nikon A1-Si) 1 day after treatment. Autofluorescence was collected with photomultiplier-type detectors at wavelength emission windows of 452/45 nm (for the 488 nm laser to detect EGFP) and 525/50 nm (for the 488 nm laser to detect Alexa Fluor 555). Images were recorded with pixel dimensions of 0.05 µm. The step size was adjusted to 0.1 μm to create Z-stack images; 96 slices were captured using a depth of 9.6 µm. Raw imaging data were edited using NIS Elements software (Nikon).

### Microstructure analysis

The right femurs were extracted from 9-week-old osteoblast-specific deletion of miR-143/145 (miR-143/145_Osx_^−/−^) and floxed (miR-143/145^flox/+^) male mice (body weight: 20–24 g). A cone-beam X-ray micro-CT system (ScanXmate-RB090SS150; Comscantecno) was used to obtain CT images of the distal ends of femurs. The settings were as follows: tube voltage, 70 kV; tube current, 0.1 mA; and voxel size, 12.0 µm. The 3D images were reconstructed and analyzed using TRI/3D-BON software (RATOC System Engineering). Regions of interest were drawn 50 µm from the end of each epiphyseal growth plate to points 0.5 mm along the cortical wall.

### Bone morphometric analysis

The left femurs were extracted from 9-week-old osteoblast-specific deletion of miR-143/145 (miR-143/145_Osx_^−/−^) and floxed (miR-143/145^flox/+^ or miR-143/145^flox/+^) male mice and were performed bone morphometric analysis as described previously^49^. For toluidine blue staining, femurs were excised, fixed in 100% ethanol, and embedded in glycolmethacrylate. For double staining of cortical bone surfaces, mice were injected subcutaneously with calcein (8 mg/kg) at 4 days or 1 day before being sacrificed.

### Phenotype of osteoblast-specific deletion of miR-143/145 in vitro

Primary osteoblasts from 9-week-old miR-143/145_Osx_^−/−^ and littermate floxed (miR-143/145^flox/flox^) male mice were analyzed. RNA extraction was performed 10 days after starting osteoblast differentiation. The expression of miR-143-3p in primary osteoblasts cultured for 14 days was assessed.

### PTH administration in vivo

Intermittent PTH injections were conducted to 5-week-old female WT and miR-143/145_Osx_^−/−^ mice (PTH: human PTH (1–34) [teriparatide; Asahi Kasei Pharma Corporation]). The dose of human PTH (1–34) was 40 µg/kg body weight per day for 5 days per week, delivered via subcutaneous injection for 4 weeks, as described previously^36^. Micro-CT analysis, bone morphometric analysis, and measurement of the urine C-terminal telopeptide-1 (CTX-1) level were performed after PTH administration at 9 weeks of age (body weight 18–22 g). The right femurs were extracted for micro-CT analysis, and the left femurs were used for bone morphometric analyses with Villanueva bone staining embedded in methylmethacrylate as described above. For measurement of CTX-1, mice were fasted for 12 h before urine sampling by bladder puncture. CTX-1 and creatinine levels in the urine samples were measured by enzyme-linked immunosorbent assay (RatLaps TM; Immunodiagnostics Systems) and enzymatic method (Wako), respectively. The CTX-1 level was corrected for the creatinine concentration.

### SOV transplantation on calvarial bone defects

C57BL/6 J male mice were anesthetized with isoflurane, and an incision was made on the head skin to expose the calvaria. Artificial calvarial defects were created on both sides using surgical punches and needles. Round defects with diameters of 1.0 mm were created for comparison between the total SOV-treated group and PBS-treated group; round defects with diameters of 1.5 mm were created for comparison of the effect of total SOVs from miR-143/145 KO mice with the effect of total SOVs from WT mice. A gelatin hydrogel sheet (MedGel II PI9, Nitta-gelatin, Japan) was cut to the same size as the defect and impregnated with PBS or SOVs (1–5 × 10^9^). The hydrogels were transplanted into either side of the calvaria defect. A micro-CT assessment was performed after 8 weeks (for the PBS- and total SOV-treated groups) or 2 weeks (for total SOVs from WT and total SOVs from miR-143/145 KO mice). Defect areas were measured using ImageJ.

### Treatment of miR-143/145-deleted SOVs in vitro

Primary osteoblasts from WT and miR-143/145_Osx_^−/−^ female mice were cultured for 14 days. SOVs were collected and named WT-SOV and KO-SOV, respectively. Primary osteoblasts from WT female mice were cultured for 6 days and treated with PBS, WT-SOV or KO-SOV. RNA extraction was performed 2 days after treatment.

### Statistics and reproducibility

Differences between two groups were analyzed using two-tailed paired, unpaired, or Welch’s *t*-tests. Differences among three or more groups were analyzed by one-way analysis of variance (ANOVA) with Dunnett’s multiple comparison *post hoc* test. Welch’s ANOVA followed by Bonferroni’s multiple comparisons test was used when the data demonstrated unequal variance. Statistical analyses were performed using Prism ver. 6 (GraphPad Software) and JMP16. Numbers of samples and animals are indicated in the figure legends. All data are representative of those of at least three independent experiments unless otherwise indicated. Biological replicates comprised samples from different mice. We estimated the required sample sizes by considering variations and means, and sought to reach reliable conclusions using sample sizes that were as small as possible.

### Reporting summary

Further information on research design is available in the Nature Research Reporting Summary linked to this article.

## Supplementary information

Supplementary Information

Reporting Summary

Description of Additional Supplementary Files

Supplementary video 1

Supplementary video 2

Supplementary video 3

Supplementary video 4

Supplementary video 5

## Data Availability

The RNA-seq data of SOVs from primary mouse osteoblasts have been deposited in the NCBI Gene Expression Omnibus (GEO) database under accession number and hyperlinks: GSE144512. All other data that support the findings of this study are available from the corresponding author upon reasonable request. Source data are provided with this paper.

## Source data

Source data
