# Supplementary material for: Osteoblast-derived vesicles induce a switch from bone-formation to bone-resorption in vivo
Source: Nat Commun. 2022 Feb 24;13:1066. doi: 10.1038/s41467-022-28673-2 (PMC8873258; doi:10.1038/s41467-022-28673-2)
Supplement: Supplementary file 1 — Supplementary Information [file 41467_2022_28673_MOESM1_ESM.pdf]

Supplementary Information

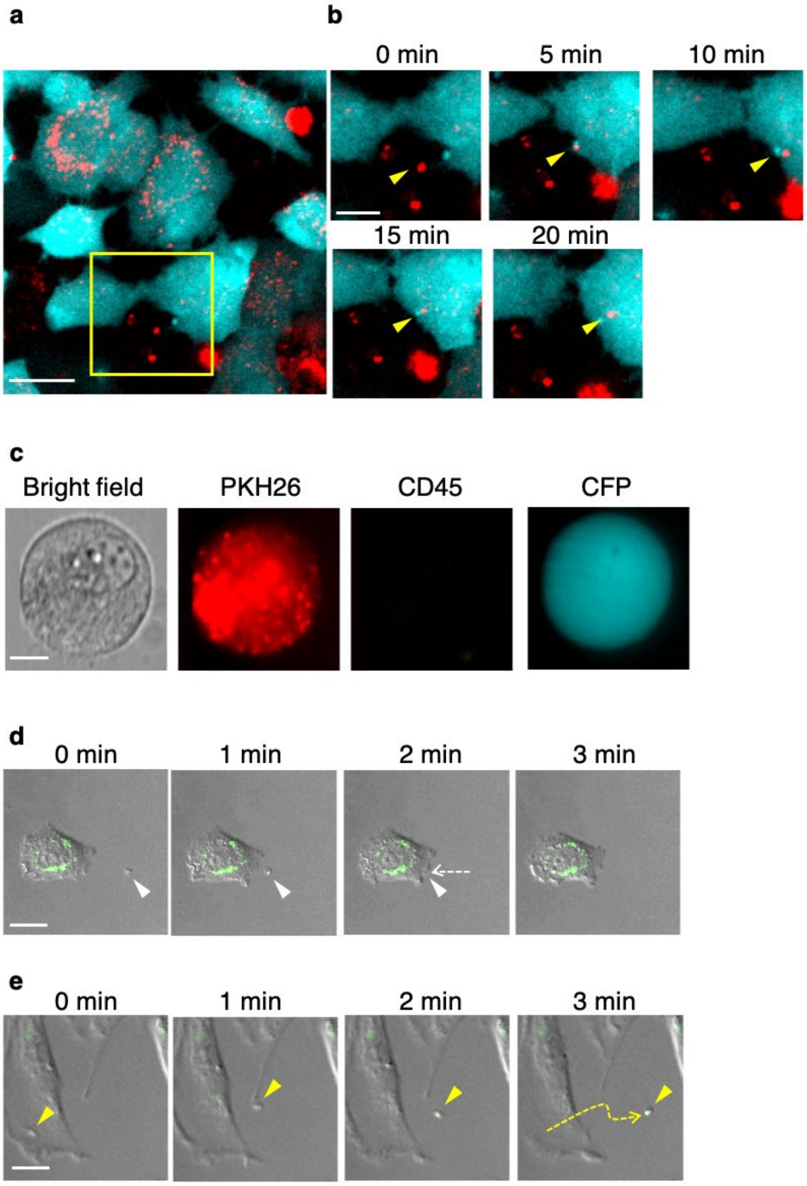

Supplementary Fig. S1. Communication among osteoblasts via small osteoblast vesicles *in vitro*.

a–c, Visualization of PKH-labeled SOV uptake *in vitro*. (**a**) A representative image of primary osteoblasts from Col2.3-ECFP mice treated with PKH-labeled SOVs

isolated from mOBs (at least three independent experiments). Red: PKH-labeled SOVs; cyan: mOBs. Scale bars: 20 μm. (**b**) Uptake of PKH-labeled SOVs by mOBs. Consecutive time-lapse images in yellow lines in (**a**). Arrowheads represent PKH-labeled SOVs taken up by mOBs. Scale bars: 10 μm. (**c**) Representative flow cytometry images. Primary osteoblasts treated with PKH26-labeled SOVs expressed CFP and PKH26, but they did not express CD45. Scale bar: 10 μm. **d, e,** Visualization of the uptake (**d**) and release (**e**) of CD63-EGFP-positive SOVs by CD63-EGFP-expressing MC3T3-E1 cells (at least three independent experiments). Green: CD63-EGFP. Arrowheads and dotted lines represent CD63-EGFP-positive SOVs and their tracks, respectively. Scale bar: 20 μm.

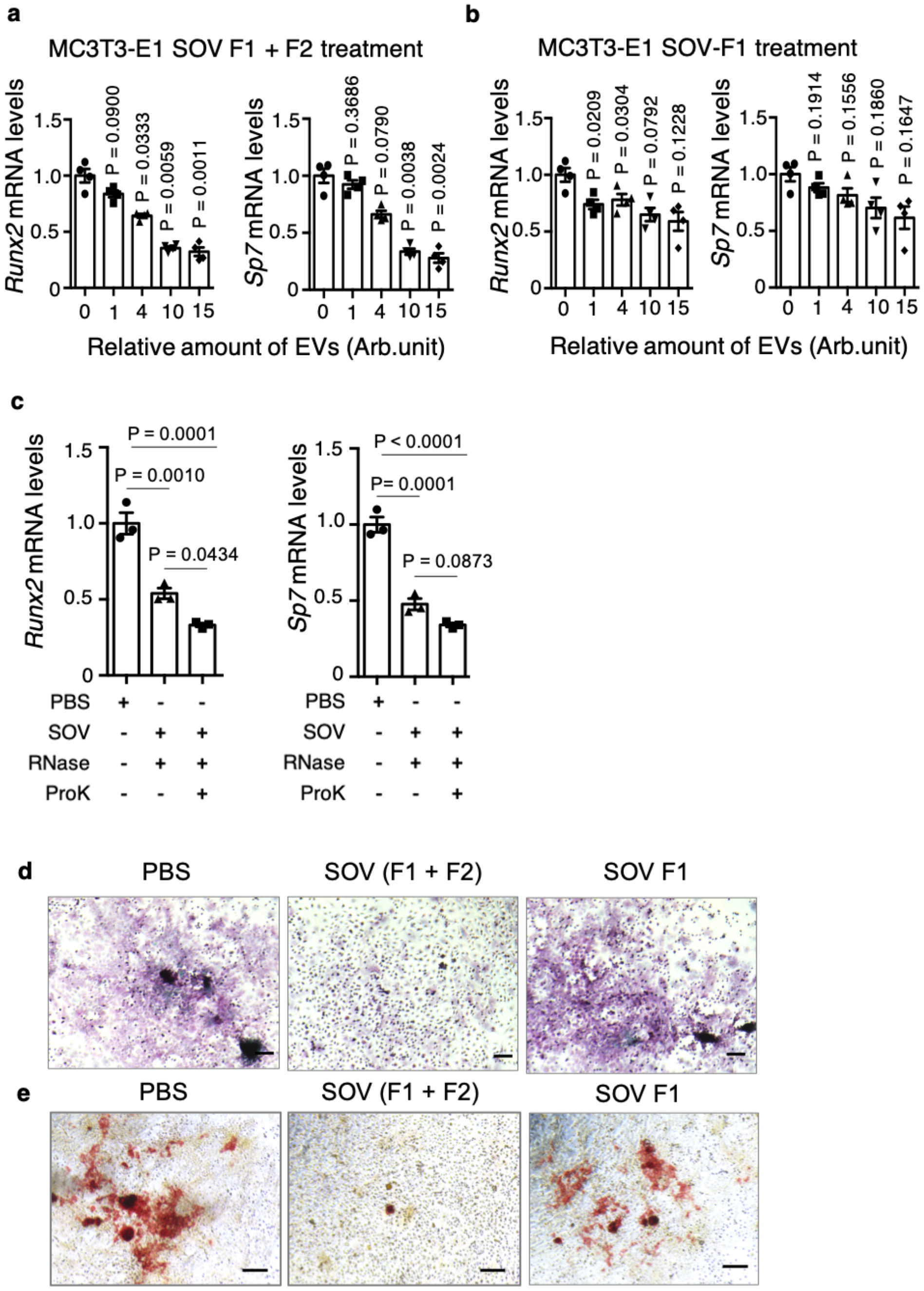

Supplementary Fig. S2. SOV-F2 inhibits osteoblast differentiation *in vitro*.

a, b, *Runx2* and *Sp7* mRNA levels in MC3T3-E1 cells treated with total SOVs (F1 + F2) (**a**) or SOV-F1 (**b**) (n = 4 independent experiment per group). **c,** Effect of RNase and proteinase K (ProK)-treated SOVs on osteoblast differentiation.

Runx2, and *Sp7* mRNA levels in osteoblasts treated with PBS, total SOVs (F1+ F2) with RNase, or total SOVs (F1 + F2) with RNase and ProK (n = 3 independent experiment per group). SOV: total SOVs (F1 + F2). **d**, Representative images of Fast Green/Sirius Red staining in osteoblasts treated with PBS, total SOVs (F1 + F2), or SOV-F1 (n = 3 independent experiments per group). Scale bar: 200 μm. **e**, Representative images of Alizarin Red S staining of osteoblasts treated with PBS, total SOVs (F1 + F2), or SOV-F1 (n = 3 independent experiments per group). Scale bar: 200 μm. Data are means ± SEMs. Statistical significance was determined by one-way ANOVA with Dunnett's multiple comparison *post hoc* test in (**a**, **b**, and **c**).

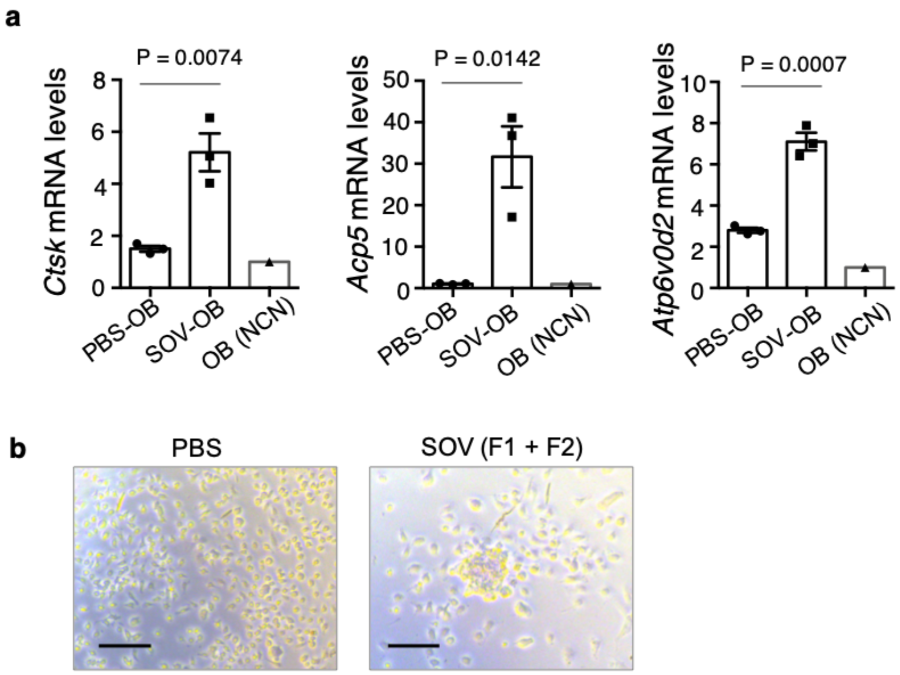

Supplementary Fig. S3. Total SOV treated osteoblasts promote osteoclast differentiation *in vitro*.

a, Mature osteoclast (mOC) marker genes (*Ctsk, Acp5,* and *Atp6v0d2*) mRNA levels in BMMs co-cultured with PBS-treated osteoblasts (PBS-OB) or BMMs co-cultured with total SOV-treated osteoblasts (SOV-OB) (n = 3 biological replicates per group). Expression levels of mOC marker genes in primary osteoblast culture as a negative control [OB(NCN)] (n = 1). **b**, Representative images of TRAP staining of BMMs with PBS or BMMs with total SOV (F1 + F2) for 3 days on macrophage colony-stimulating factor (M-CSF) (n = 8 independent experiments per group). There were no significant positive areas in either group. Bar = 100 µm. Data are means ± SEMs. Statistical significance was determined by two-tailed unpaired *t*-test in (**a**).

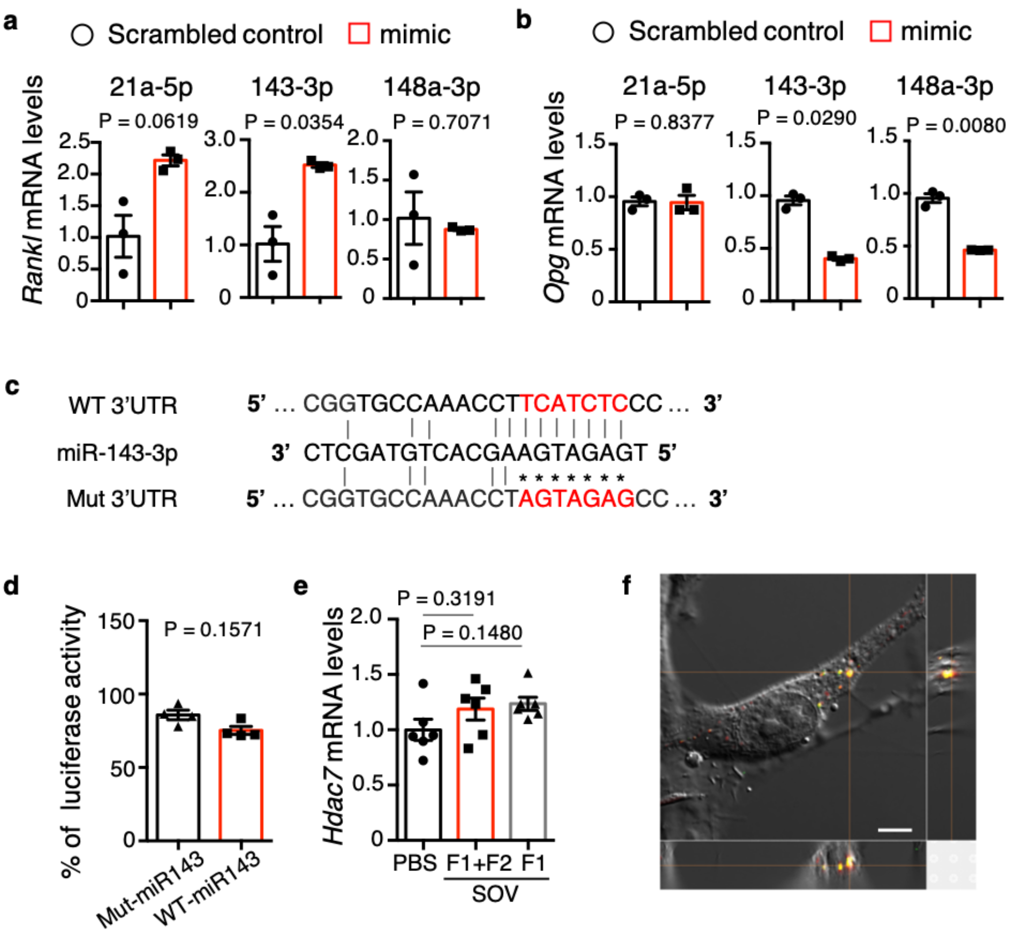

Supplementary Fig. S4. Effects of miR-143-3p on osteoblasts *in vitro*.

a, b, *Rankl* (**a**) and *Opg* (**b**) mRNA levels in MC3T3-E1 cells transfected with scrambled control, miR-21a-5p, miR-143-3p, or miR-148a-3p mimics (n = 3 independent experiment per group). **c–e**, Effect of miR-143-3p in mature osteoblast-derived SOVs on *Hdac7* expression. The *Hdac7* 3′ UTR was cloned into luciferase reporter vectors. WT 3′ UTR: WT sequence of the *Hdac7* 3′ UTR; miR-143-3p: miR-143-3p sequence; Mut 3′ UTR: mutated sequence of the *Hdac7* 3′ UTR (**c**), percentages of luciferase activity Mut-miR143: mutated *Hdac7* construct with miR-143-3p mimics; WT-miR143: WT *Hdac7* construct with miR-

143-3p mimics. (n = 4 independent experiment per group) (**d**), and *Hdac7* mRNA levels in primary osteoblasts treated with PBS, total SOVs (F1 + F2), or SOV-F1 (n = 6 biological replicates per group) (**e**). **f**, A representative image of MC3T3-E1 cells capturing miR-143-3p-containing SOVs (n = 4 independent experiments). Red: miR-143-3p; green: SOVs; yellow: miR-143-3p-containing SOVs. Scale bar: 5 μm. Data are means ± SEMs. Statistical significance was determined by two-tailed paired *t*-test in (**a**, **b,** and **d**) and by one-way ANOVA with Dunnett's multiple comparison *post hoc* test in (**e**).

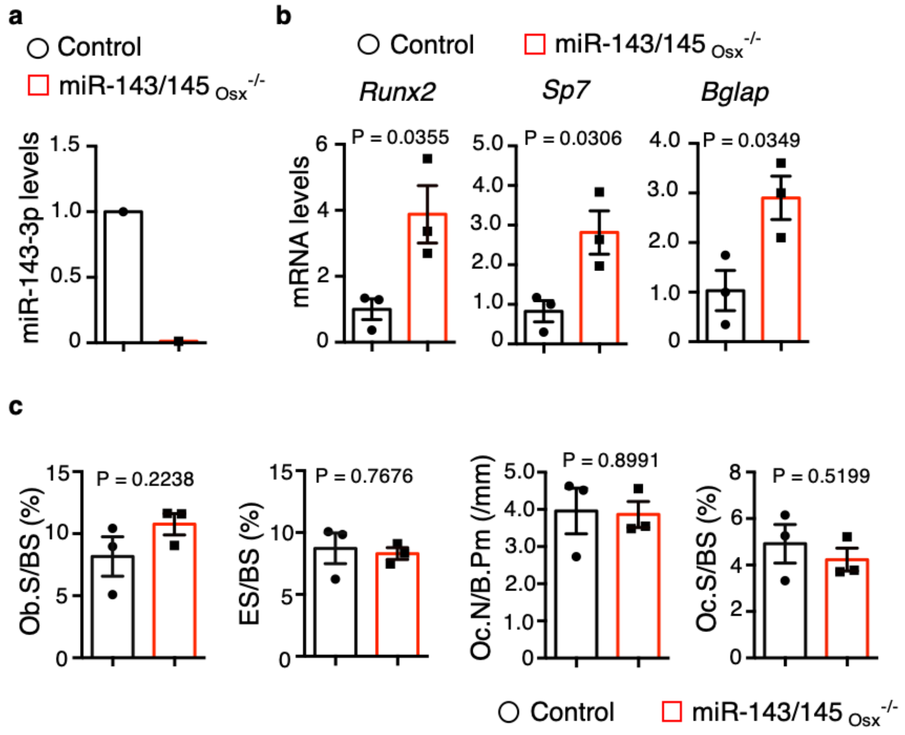

Supplementary Fig. S5. Effects of osteoblast-specific deletion of miR-143/145 in mice *in vitro* and *in vivo*.

a, Expression levels of miR-143-3p in primary osteoblasts of 9-week-old control and osteoblast-specific miR-143/145 KO (miR-143/145$_{Osx}$$^{-/-}$) mice (n = 1 from 3 mice). **b,** *Runx2*, *Sp7*, and *Bglap* mRNA levels in primary osteoblasts of 9-week-old control and miR-143/145$_{Osx}$$^{-/-}$ mice (n = 3 biological replicates per group). **c,** Bone morphometric analysis of metaphyseal regions of distal femurs of 9-week-old control and miR-143/145$_{Osx}$$^{-/-}$ mice (n = 3 biological replicates per group). BS: bone surface, Ob.S: osteoblast surface; ES: eroded surface; Oc.N: osteoclast number; Oc.S: osteoclast surface; B.Pm: bone perimeter. Data are means ± SEMs. Statistical significance was determined by two-tailed unpaired *t*-test in (**b**) and (**c**).

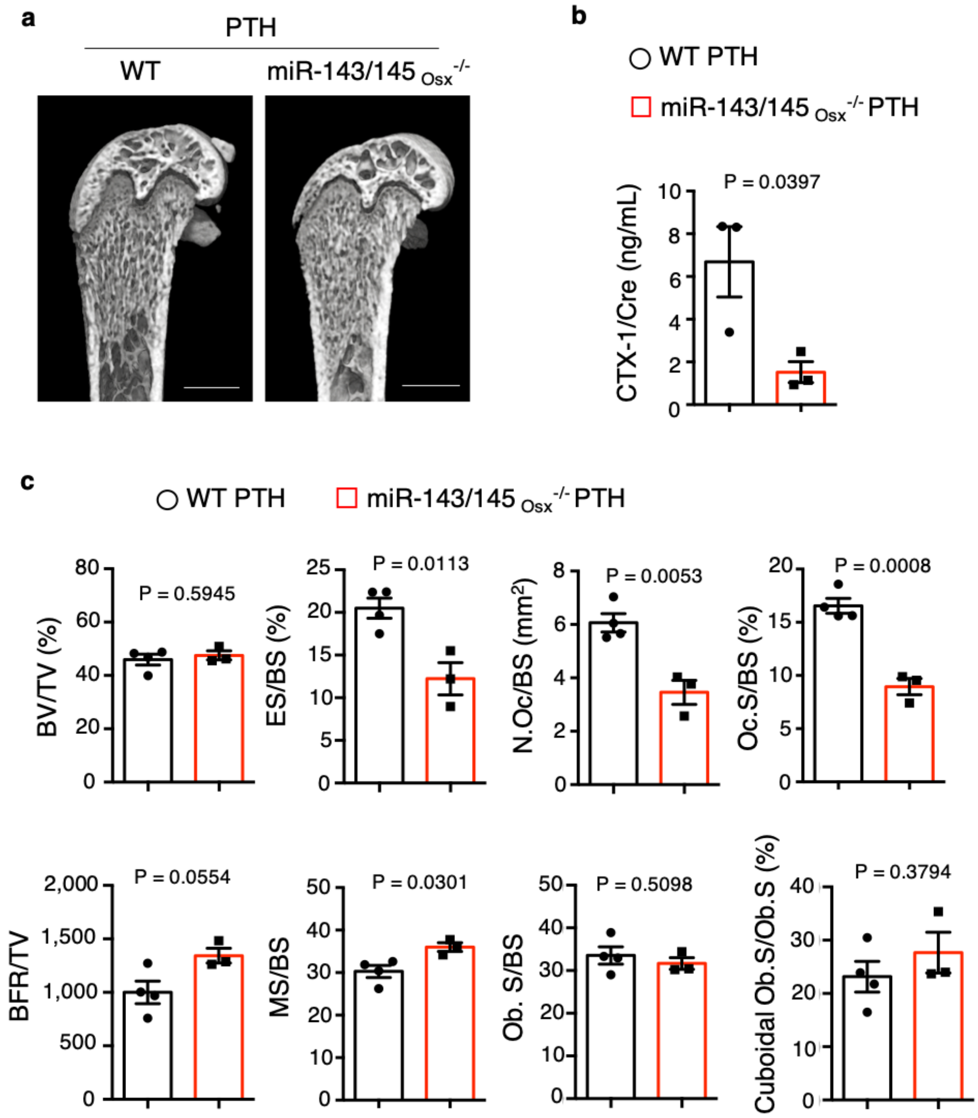

Supplementary Fig. S6. miR-143/145 conditional knockout decreases osteoclast activity after parathyroid hormone administration *in vivo.*

a, Representative micro-CT images of the femurs of 9-week-old wild-type (WT) (n = 4 biological replicates) and osteoblast-specific miR-143/145 KO (miR-143/145$_{Osx}^{-/-}$) mice (n = 3 biological replicates) after intermittent parathyroid hormone (PTH) injections. Scale bars: 1000 μm. **b**, Urine CTX-1 levels (corrected

for urine creatinine) in PTH-treated WT mice (WT PTH) and PTH-treated miR-143/145$_{Osx}^{-/-}$ mice (miR-143/145$_{Osx}^{-/-}$ PTH) (n = 3 biological replicates per group). **c**, Bone morphometric analyses of metaphyseal regions of distal femurs of 9-week-old PTH-treated WT (n = 4 biological replicates) and PTH-treated miR-143/145$_{Osx}^{-/-}$ mice (n = 3 biological replicates). BV: bone volume; TV: tissue volume; ES: eroded surface; BS: bone surface; N.Oc: osteoclast number; Oc.S: osteoclast surface. Data are means ± SEMs. Statistical significance was determined by two-tailed unpaired *t*-test in (**b** and **c**).

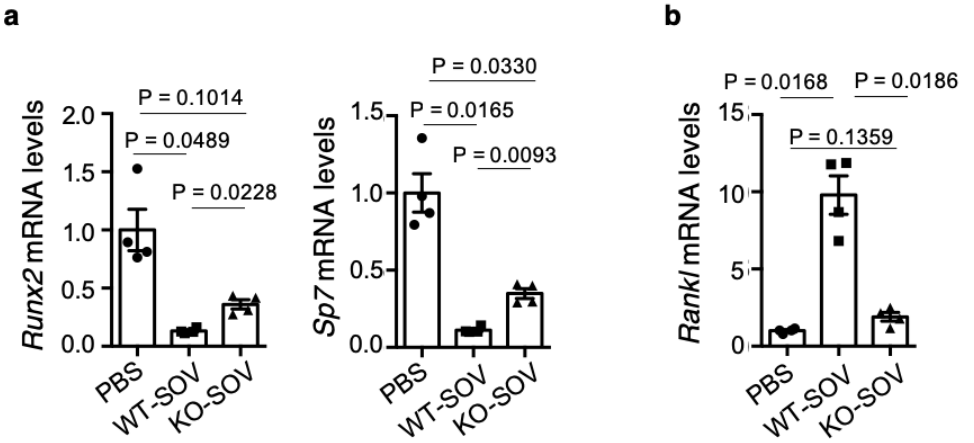

Supplementary Fig. S7. MiR-143/145-deleted SOVs reverse the effects of SOVs on osteoblasts.

a, b, Reversal of SOV effects on osteoblasts by miR-143/145-deleted SOVs. *Runx2*, *Sp7* (**a**), and *Rankl* (**b**) mRNA levels in primary osteoblasts treated with PBS, WT-derived SOVs (WT-SOV), or miR-143/145$_{Osx}^{-/-}$ derived SOVs (KO-SOV) (n = 4 biological replicates per group). Data are means ± SEMs. Statistical significance was determined by Welch's ANOVA followed by *post hoc* two-tailed Welch's *t*-test with Bonferroni correction in (**a** and **b**).

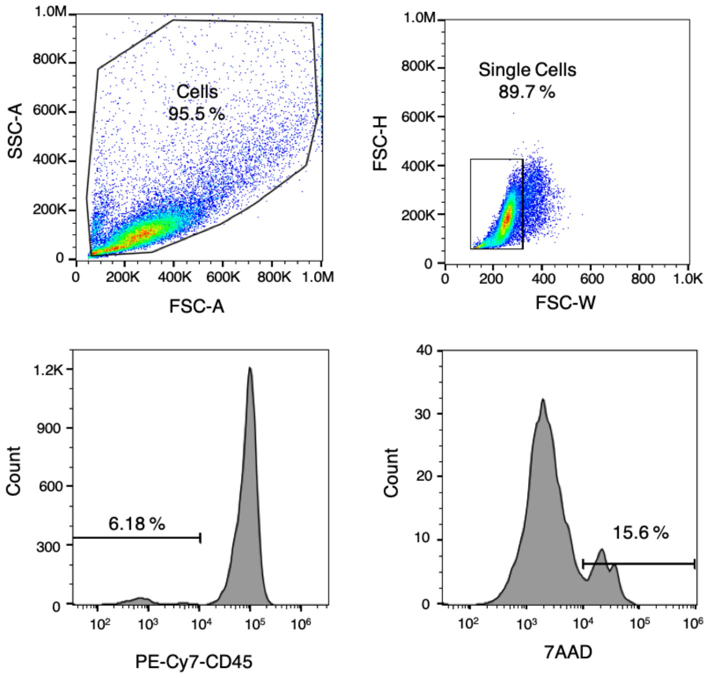

Supplementary Fig. S8. FACS gating strategy.

Gating strategies for the evaluation of apoptotic osteoblasts with 7AAD after the treatment of SOVs (Fig. 2).

Supplementary Table 1: Sequences of oligonucleotide of primers for qPCR and the inserted sequences for the luciferase assay in this study.

	Forward (5' → 3')	Revers (5' → 3')
Actb	CTTCTACAATGAGCTGCGTG	TCATGAGGTAGTCTGTCAGG
Runnx2	AGGCACAAAGAAGCCATAC	AATGAGTGAGGGAAGGGT
Sp7	CATCTGCCTGACTCCTTGGGAC	GCTGAAAGGTCAGCGTATGGC
Alpl	CCCAAGGAAAAGAAGCACGTC	ACATTAGGCGCAGGAAGGTCA
Col1a1	TGGAAGAGCGGAGAGTACTG	GATAGGTGATGTTCTGGGAGG
Bglap	TGGCGACACTTACCGAGCTT	CCATGCCCCTTGTAGTAGCTGTA
Rankl	CAGCATCGCTCTGTTCCTGTA	CTGCGTTTTCATGGAGTCTCA
Opg	GTTTCCCGAGGACCACAAT	CCATTCAATGATGTCCAGGAG
Cbfb	TGTGAGATTAAGTACACGG	TAATGCATCCTCCTGCTGGGCT
Hdac7	TGAAGAATGGCTTTGCTGTG	CACTGGGGTCCTGGTAGAAA
Ctsk	GAAGAAGACTCACCAGAAGCAG	TCCAGGTTATGGGCAGAGATT
Acp5	CACTCCCACCCTGAGATTTGT	CATCGTCTGCACGGTTCTG
Atp6v0d2	CAGAGCTGTACTTCAATGTGGAC	AGGTCTCACACTGCACTAGGT

	5' → 3'
Cbfb-sense	CTAGCGGCCGCTAGTCATCATTGCATCATTTTTTAAAGATTC ATCTCCATTAAAACTTGCCTTAAGCTTCCT
Cbfb-antisense	CTAGAGGAAGCTTAAGGCAAGTTTTAATGGAGATGAATCTT TAAAAAATGATGCAATGATGACTAGCGGCCGCTAGAGCT
Cbfb mutant-sense	CTAGCGGCCGCTAGTCATCATTGCATCATTTTTTAAAGATAG TAGAGCATTAAAACTTGCCTTAAGCTTCCT
Cbfb mutant-antisense	CTAGAGGAAGCTTAAGGCAAGTTTTAATGCTCTACTATCTTT AAAAAATGATGCAATGATGACTAGCGGCCGCTAGAGCT
Hdac7-sense	CTAGCGGCCGCTAGTCTCCTAACCCAACGGTGCCAAACCT TCATCTCCCTTCAAAAGCACAACACAATCCCT
Hdac7-antisense	CTAGAGGGATTGTGTTGTGCTTTTGAAGGGAGATGAAGGTT TGGCACCGTTGGGTTAGGAGACTAGCGGCCGCTAGAGCT
Hdac7 mutant-sense	CTAGCGGCCGCTAGTCTCCTAACCCAACGGTGCCAAACCT AGTAGAGCCTTCAAAAGCACAACACAATCCCT
Hdac7 mutant -antisense	CTAGAGGGATTGTGTTGTGCTTTTGAAGGCTCTACTAGGTT TGGCACCGTTGGGTTAGGAGACTAGCGGCCGCTAGAGCT
