# Supplementary material for: Osteoblast-derived vesicles induce a switch from bone-formation to bone-resorption in vivo
Source: Nat Commun. 2022 Feb 24;13:1066. doi: 10.1038/s41467-022-28673-2 (PMC8873258; doi:10.1038/s41467-022-28673-2)
Supplement: Supplementary file 2 — Reporting Summary [file 41467_2022_28673_MOESM2_ESM.pdf]

nature research

Corresponding author(s): Junichi Kikuta and Masaru Ishii

Last updated by author(s): Dec 8, 2021

Reporting Summary

Nature Research wishes to improve the reproducibility of the work that we publish. This form provides structure for consistency and transparency in reporting. For further information on Nature Research policies, see our Editorial Policies and the Editorial Policy Checklist.

Statistics

For all statistical analyses, confirm that the following items are present in the figure legend, table legend, main text, or Methods section.

n/a	Confirmed	
☐	☒	The exact sample size (n) for each experimental group/condition, given as a discrete number and unit of measurement
☐	☒	A statement on whether measurements were taken from distinct samples or whether the same sample was measured repeatedly
☐	☒	The statistical test(s) used AND whether they are one- or two-sided<br>*Only common tests should be described solely by name; describe more complex techniques in the Methods section.*
☒	☐	A description of all covariates tested
☐	☒	A description of any assumptions or corrections, such as tests of normality and adjustment for multiple comparisons
☐	☒	A full description of the statistical parameters including central tendency (e.g. means) or other basic estimates (e.g. regression coefficient) AND variation (e.g. standard deviation) or associated estimates of uncertainty (e.g. confidence intervals)
☐	☒	For null hypothesis testing, the test statistic (e.g. F, t, r) with confidence intervals, effect sizes, degrees of freedom and P value noted<br>*Give P values as exact values whenever suitable.*
☒	☐	For Bayesian analysis, information on the choice of priors and Markov chain Monte Carlo settings
☒	☐	For hierarchical and complex designs, identification of the appropriate level for tests and full reporting of outcomes
☒	☐	Estimates of effect sizes (e.g. Cohen's d, Pearson's r), indicating how they were calculated

Our web collection on statistics for biologists contains articles on many of the points above.

Software and code

Policy information about availability of computer code

Data collection	NIS Elements v.4.50.00, HiSeq 2500 platform, CLC genomics workbench v9.5.3, H-7650, Nanosight LM10, TP800 Dice Real-Time Thermal Cycler System, A cone-beam X-ray micro-CT system, Luminescent Micro Plate Reader
Data analysis	FlowJo v.10, IDEAS v.6.0, NTA2.3 software, GraphPad Prism v.6, JMP16, Imaris v.8.3.1, Adobe After Effects CS6, ImageJ 1.53a with Java 1.8.0, TRI/3D-BON software

For manuscripts utilizing custom algorithms or software that are central to the research but not yet described in published literature, software must be made available to editors and reviewers. We strongly encourage code deposition in a community repository (e.g. GitHub). See the Nature Research guidelines for submitting code & software for further information.

Data

Policy information about availability of data

All manuscripts must include a data availability statement. This statement should provide the following information, where applicable:

- Accession codes, unique identifiers, or web links for publicly available datasets
- A list of figures that have associated raw data
- A description of any restrictions on data availability

The RNA-seq data of SOVs from primary mouse osteoblasts have been deposited in the NCBI Gene Expression Omnibus (GEO) database under accession number and hyperlinks: GSE144512 [https://www.ncbi.nlm.nih.gov/geo/query/acc.cgi?acc= GSE144512]. Source data underlying all Figures are provided as a Source Data file. All other data that support the findings of this study are available from the corresponding author upon reasonable request.

Field-specific reporting

Please select the one below that is the best fit for your research. If you are not sure, read the appropriate sections before making your selection.

- [x] Life sciences
- [ ] Behavioural & social sciences
- [ ] Ecological, evolutionary & environmental sciences

For a reference copy of the document with all sections, see nature.com/documents/nr-reporting-summary-flat.pdf

Life sciences study design

All studies must disclose on these points even when the disclosure is negative.

Sample size	We estimated the required sample sizes by considering variations and means, and sought to reach reliable conclusions using sample sizes that were as small as possible. Previously published results, complexity, the cost of experiments and past experience were used to determine the sample size.
Data exclusions	No data were excluded.
Replication	Experiments included sufficient sample size to ensure the reproducibility of the findings. Representative data was confirmed at least once with an independent experiment. All attempts at replication were successful.
Randomization	For in vivo studies, mice were randomly assigned to each treatment groups within each genotype. For in vitro studies, conditions were randomly assigned to each experimental condition.
Blinding	Assays were not relevant to blinding when the same author conducted the experiments and analyzed them. The majority of our experiments were not blinded except for bone histomorphometric analysis of osteoblast specific miR-143/145 KO male mice. In the analysis, investigators were blinded, because the analysis was outsourcing.

Reporting for specific materials, systems and methods

We require information from authors about some types of materials, experimental systems and methods used in many studies. Here, indicate whether each material, system or method listed is relevant to your study. If you are not sure if a list item applies to your research, read the appropriate section before selecting a response.

Materials & experimental systems

n/a	Involved in the study	
☐	☒	Antibodies
☐	☒	Eukaryotic cell lines
☒	☐	Palaeontology and archaeology
☐	☒	Animals and other organisms
☒	☐	Human research participants
☒	☐	Clinical data
☒	☐	Dual use research of concern

Methods

n/a	Involved in the study	
☒	☐	ChIP-seq
☐	☒	Flow cytometry
☒	☐	MRI-based neuroimaging

Antibodies

Antibodies used	anti-mouse CD45 PE-Cy7 (supplier: BioLegend, Cat.103114, Clone: 30-F11). 1:100 dilution for flow cytometry.
Validation	The anti-mouse CD45 PE-Cy7 antibody is commercially available and their validation statements are available on the manufacturer's website. https://www.biolegend.com/ja-jp/search-results/pe-cyanine7-anti-mouse-cd45-antibody-1903

Eukaryotic cell lines

Policy information about cell lines

Cell line source(s)	MC3T3-E1 (CRL-2593TM), HEK293T (CRL-3216TM) from ATCC, Manassas, VA, USA
Authentication	We checked the cell lines used in this study against the list of known misidentified cell lines maintained by International Cell Line Authentication Committee.
Mycoplasma contamination	Not tested
Commonly misidentified lines (See ICLAC register)	No commonly misidentified lines

Animals and other organisms

Policy information about studies involving animals; ARRIVE guidelines recommended for reporting animal research

Laboratory animals	C57BL6/J mice(sex: male and female ; age: 10-20 weeks and 9 weeks, respectively); Col2.3-ECFP mice (sex: male; age: 10-20 weeks); Col2.3-ECFP and TRAP-tdTomato transgenic mice (sex: male; age: 10-20 weeks); miR-143/145 floxed mice (sex: male; age: 9 weeks); miR-143/145 knockout mice (sex: male; age: 10-20 weeks); miR-143/145Osx-/- mice (sex: male and female; age: 9 weeks); Osx1-GFP::Cre: B6.Cg-Tg(Sp7-tTA,tetO-EGFP/cre)1Amc/J mice and TRAP-tdTomato transgenic mice were used for mating.
Wild animals	No wild animals were used in this study
Field-collected samples	No field collected samples were used in this study.
Ethics oversight	Animal studies were approved by the Institutional Review Board of Osaka University.

Note that full information on the approval of the study protocol must also be provided in the manuscript.

Flow Cytometry

Plots

Confirm that:

- [x] The axis labels state the marker and fluorochrome used (e.g. CD4-FITC).
- [x] The axis scales are clearly visible. Include numbers along axes only for bottom left plot of group (a 'group' is an analysis of identical markers).
- [x] All plots are contour plots with outliers or pseudocolor plots.
- [x] A numerical value for number of cells or percentage (with statistics) is provided.

Methodology

Sample preparation	Cells were collected by centrifugation at 300 g after treatment with 0.2% collagenase P (Roche) for 1 h at 37°C, followed by 0.25% trypsin (containing 0.02% EDTA) for 10 min. For the analysis of cells after PKH labeled SOV treatment, Single-cell suspensions (2 × 107/mL) were incubated with PE-Cy7 conjugated anti-mouse CD45 antibody (BioLegend) diluted in FACS buffer at 1:100 ratio for 15 min. Flow cytometric data were analyzed using IDEAS software (Amnis, EMD Millipore).<br>For the analysis of dead cells, Single-cell suspension (1 × 106/mL) were incubated with CD45-PECy7 (BioLegend) diluted in FACS buffer at 1:100 ratio for 15 min, and dead cells were stained with 10 μg/m 7-AAD (BD Biosciences) immediately before analysis. Flow cytometry data were analyzed using FlowJo software (TreeStar).
Instrument	Cell sorter SH800 (SONY), ImageStreamX Mark II cytometer (Amnis, EMD Millipore)
Software	FlowJo v.10, IDEAS v.6.0
Cell population abundance	The abundance of positive cell population was generally above 5%.
Gating strategy	The stained samples were matched with non-stained negative controls to determine the positive and negative population. In FSC/SSC chart, we removed cell debris and gated cell-population. Cells were gated for singlet according to FSC-H/FSC-A. The gated cells were then gated for each desired positive or negative cells according to each determination.

- [x] Tick this box to confirm that a figure exemplifying the gating strategy is provided in the Supplementary Information.
