# Supplementary material for: Osteoblast-derived vesicles induce a switch from bone-formation to bone-resorption in vivo
Source: Nat Commun. 2022 Feb 24;13:1066. doi: 10.1038/s41467-022-28673-2 (PMC8873258; doi:10.1038/s41467-022-28673-2)
Supplement: Supplementary file 3 — Description of Additional Supplementary Files [file 41467_2022_28673_MOESM3_ESM.pdf]

**Title:** Supplementary Video 1.
**Description:** Intravital multiphoton imaging of skull bone tissues from Col2.3-ECFP mice. Left: an original video; cyan: mOBs and SOVs; blue: bone tissue, second harmonic generation. Right: a schematic video; cyan: mOBs; yellow balls: SOVs. mOBs are releasing and taking up SOVs. Scale bar: 5 µm. Playback speed = 1800×.

**Title:** Supplementary Video 2.
**Description:** In vitro imaging of primary osteoblasts releasing SOVs. Cyan: mOBs and SOVs. mOBs are releasing SOVs. Scale bar: 10 µm. Playback speed = 600×.

**Title:** Supplementary Video 3.
**Description:** In vitro imaging of primary osteoblasts taking up SOVs. Cyan: mOBs and SOVs. mOBs are taking up SOVs. Scale bar: 10 µm. Playback speed = 600×.

**Title:** Supplementary Video 4.
**Description:** In vitro imaging of primary osteoblasts from Col2.3-ECFP mice treated with PKHlabeled SOVs. Cyan: mOBs; red: PKH-labeled SOVs. mOBs are taking up PKH-labeled SOVs. Scale bar: 10 µm. Playback speed = 600×.

**Title:** Supplementary Video 5.
**Description:** In vitro imaging of primary osteoblasts from Col2.3-ECFP mice treated with PKHlabeled SOVs. Cyan: mOBs; red: PKH-labeled SOVs. mOBs are taking up PKH-labeled SOVs. Scale bar: 10 µm. Playback speed = 600×.
